# *Xmrk*, *Kras* and *Myc* Transgenic Zebrafish Liver Cancer Models Share Molecular Signatures with Subsets of Human Hepatocellular Carcinoma

**DOI:** 10.1371/journal.pone.0091179

**Published:** 2014-03-14

**Authors:** Weiling Zheng, Zhen Li, Anh Tuan Nguyen, Caixia Li, Alexander Emelyanov, Zhiyuan Gong

**Affiliations:** 1 Department of Biological Sciences, National University of Singapore, Singapore, Singapore; 2 Institute of Cell and Molecular Biology, Singapore, Singapore; Mayo Clinic, United States of America

## Abstract

Previously three oncogene transgenic zebrafish lines with inducible expression of *xmrk*, *kras* or *Myc* in the liver have been generated and these transgenic lines develop oncogene-addicted liver tumors upon chemical induction. In the current study, comparative transcriptomic approaches were used to examine the correlation of the three induced transgenic liver cancers with human liver cancers. RNA profiles from the three zebrafish tumors indicated relatively small overlaps of significantly deregulated genes and biological pathways. Nevertheless, the three transgenic tumor signatures all showed significant correlation with advanced or very advanced human hepatocellular carcinoma (HCC). Interestingly, molecular signature from each oncogene-induced zebrafish liver tumor correlated with only a small subset of human HCC samples (24–29%) and there were conserved up-regulated pathways between the zebrafish and correlated human HCC subgroup. The three zebrafish liver cancer models together represented nearly half (47.2%) of human HCCs while some human HCCs showed significant correlation with more than one signature defined from the three oncogene-addicted zebrafish tumors. In contrast, commonly deregulated genes (21 up and 16 down) in the three zebrafish tumor models generally showed accordant deregulation in the majority of human HCCs, suggesting that these genes might be more consistently deregulated in a broad range of human HCCs with different molecular mechanisms and thus serve as common diagnosis markers and therapeutic targets. Thus, these transgenic zebrafish models with well-defined oncogene-induced tumors are valuable tools for molecular classification of human HCCs and for understanding of molecular drivers in hepatocarcinogenesis in each human HCC subgroup.

## Introduction

Human hepatocellular carcinoma (HCC) is known to be a very heterogeneous disease, especially at intermediate and advanced stages [1]. Due to the diverse and complex etiologies contributing to HCC incidence, different genetic mutations or altered molecular pathways could be responsible for hepatocarcinogenesis. So far, several carcinogenic pathways have been identified to be involved in the development and progression of HCC, including the VEGFR, EGFR, and mTOR pathways [2]. In effort to decipher the role of different oncogenic pathways, a number of transgenic mouse models have been established [3], [4] and comparative transcriptomic analyses have been used to identify the best transgenic mouse models for human HCCs [4].

The zebrafish has been increasingly recognized as a valuable experimental model for human diseases, particularly for cancers [5] including liver cancers [6]–[11]. It has been shown that the zebrafish tumors had striking similarities with human cancer histologically [12], [13]. Transcriptomic and epigenetic analyses have also indicated conserved features of carcinogen-induced zebrafish HCC with human HCC [14]–[16].

Previously we have generated several liver tumor models by transgenic expression of three different oncogenes (*kras, xmrk* or *Myc*) specifically in the zebrafish liver and these transgenic zebrafish usually produce liver tumors with variable degrees of severity from hepatocellular adenoma (HCA) to HCC [6]–[9]. The three oncogenes we used in the zebrafish have all been shown to be involved in hepatocarcinogenesis. *KRAS* is mutated in ∼7% of liver cancers in human [17] but Ras signaling is ubiquitously activated in HCC [18]. *Xmrk* is a naturally occurring variant of the EGFR in fish of the genus *Xiphophorus* (platyfish and swordtails) with constitutive autophosphorylation and activation of downstream signals [19]. Activation of EGFR signal is correlated with poor prognosis of HCC patients [20]. *MYC* is commonly amplified in many cancers including HCC and higher expression level of MYC is associated with more advanced status of HCC [21]. We have shown that overexpression of *kras* and *xmrk* in the zebrafish liver could induce HCC [7]–[9], while overexpression of *Myc* induced mostly HCA [6].

Comparative transcriptomic analyses of animal models and human clinical samples provide a powerful tool for identification of conserved molecular pathways and biomarker genes for diagnosis and therapy [15], [22]. Our existing oncogene transgenic zebrafish models have well defined up-regulation of driver oncogene and this may provide a valuable tool to identify the molecular driving forces in human carcinogenesis by comparative transcriptomic analyses. Thus, in this study, we employed RNA sequencing technology to compare the transcriptomic profiles of the three oncogene-induced liver tumors in transgenic zebrafish. By comparative analyses with human liver transcriptomes from cirrhotic livers to very advanced HCC, we found that they all showed strong molecular correlation with advanced or very advanced human HCCs. Nevertheless, there are quite distinct deregulated biological pathways based on deregulated genes in the three oncogene transgenic models. Interestingly, each zebrafish liver tumor model correlated with a subset of human HCCs and each subset has some distinct molecular features. We showed that the transgenic zebrafish models with well-defined driver-gene activity should be valuable for classification of human HCCs and for understanding the molecular mechanisms behind each HCC subtype.

## Materials and Methods

### Treatment and Induction of Liver Cancer in the Three Zebrafish Transgenic Models

Zebrafish were maintained following the approved protocol by Institutional Animal Care and Use Committee of National University of Singapore (Protocol 079/07). The generation of *xmrk* and *Myc* transgenic zebrafish was previously described and they were termed as *TO(xmrk)* [9] and *TO(Myc)* [6] respectively in the previous publications. The two transgenic lines were constructed by using a tetracycline-inducible transgenic system and the oncogene expression were induced by doxycycline. The *kras^V12^* transgenic line used in the present study was newly generated by using a mifepristone-inducible system [7] in combination with a Cre-loxP system (unpublished). For the *xmrk* and *Myc* transgenic lines, transgenic fish and their non-transgenic siblings were treated with 60 µg/ml doxycycline (Sigma, USA) starting from 3.5 mpf (month post fertilization) for 6 weeks. All *xmrk* fish developed HCC and all *Myc* fish developed HCA. In total, for each transgenic line, one tumor sample (transgenic fish treated with doxycycline) and three control samples (non-transgenic siblings similarly treated with doxycycline, transgenic siblings without doxycycline treatment, and non-transgenic siblings without doxycycline treatment) were collected for RNA sequencing. In all cases, liver samples used for RNA sequencing were pooled from four to five male fish. For the *kras^V12^* transgenic fish, one-month-old transgenic fish were treated with 1 µM mifepristone (Sigma, USA) for 36 hours to induce Cre-mediated recombination for activation of *kras^V12^* transgene expression in the liver. The *kras^V12^* activated transgenic fish were then allowed to grow for six months to develop HCC and HCC samples were then collected for RNA sequencing. Three liver tumors from induced transgenic fish and three normal livers from uninduced transgenic fish were pooled separately as tumor and control samples. All samples used were from male fish and two sets of biological replicates were used.

### Identification of Signature Gene Lists in Each Zebrafish Liver Cancer Model

Total RNA was extracted using TRIzol Reagent (Invitrogen, USA) and treated with DNase I to remove genomic DNA contamination. 3′ RNA-SAGE (serial analysis of gene expression) sequencing was performed on ABI SOLiD platform by Mission Biotech (Taiwan) according to manufacturer’s protocol and 10–23 million reads were generated from each sampler (Table S1). Briefly, mRNA was purified using Dynabeads Oligo(dT) EcoP (Invitrogen) and subjected to cDNA synthesis. Resultant cDNA was digested by NlaIII and EcoP15I to result in a 27 nucleotides cDNA tag between the two sequencing adapters. The tags were mapped to the NCBI RefSeq (Reference Sequence) mRNA database for zebrafish with a criterion of allowing maximum 2 nucleotide mismatches. All RNA-Seq data were submitted to Gene Expression Omnibus database with the following access numbers: GSE53342 for *xmrk* and *Myc* data and GSE53630 for *kras* data. Tag counts for each transcript were normalized to TPM (transcripts per million) to facilitate comparison among different samples. The differentially regulated genes in the *Myc*- and *xmrk*- induced liver cancers were identified using one sample t-test as previously described [23], and the differentially expressed genes in the *kras*-induced liver cancer was identified using two-tailed Student’s t-test.

To facilitate functional implications of zebrafish transcriptome, all zebrafish genes were mapped to annotated human genes in order to use existing online software developed in human genes. Thus, human homology mapping of zebrafish Unigene clusters were retrieved from the Genome Institute of Singapore Zebrafish Annotation Database (http://123.136.65.67/). For Unigene clusters mapped by more than one transcript entries, the highest TPM was used to represent the expression level of the Unigene cluster. Some zebrafish Unigene clusters were mapped to more than one human Unigene clusters, which usually came from the same gene family. To remove redundancy and avoid causing bias in functional analyses, only the first human Unigene cluster in the list was selected to represent the zebrafish Unigene clusters. The lists of significantly up-regulated zebrafish genes that were mapped with human homologs and used for comparative analyese with human HCC data are shown in Table S2.

### Gene Set Enrichment Analysis (GSEA)

GSEA was used to establish the relatedness between the transgenic zebrafish models and human liver cancers [24]. GSEA is a computational method that determines whether a *priori* defined set of genes shows statistically significant, concordant differences between two biological samples; it calculates an enrichment score using a running-sum statistic through a ranked list of gene expression data set. Human homologs of the significantly up-regulated genes from the zebrafish tumor tissues were used as cancer signatures for each transgenic zebrafish model for transcriptomic comparison with human HCC data. Each chosen phenotype of human HCCs (either one specific HCC stage or one particular HCC sample) was compared to the rest of the samples in the same dataset. All genes in the chosen phenotype were ranked by t-test to determine expression differences among different HCC stages or different HCC samples. The enrichment score of the pre-defined transgenic zebrafish cancer signature was calculated using a running-sum statistic through the ranked genes. The statistical significance of the enrichment score was estimated by using an empirical phenotype-based permutation test procedure. An FDR (false discovery rate) value was provided by introducing adjustment of multiple hypothesis testing. Human liver cancer transcriptome data were retrieved from Gene Expression Omnibus (GEO) database. The human dataset including different stages of HCCs used in the comparison was GSE6764 [25]. The ten human HCC datasets used for examining the representation of zebrafish liver cancer gene signatures are summarized in Table S3. Annotation information was retrieved from SOURCE (http://smd.stanford.edu/cgi-bin/source/sourceSearch). For multiple probes which can be mapped to one Unigene cluster, the maximum signal intensity was selected to represent the expression level of the Unigene cluster.

### GSEA Pre-ranked Analysis

GSEA pre-ranked option was used to analyze the deregulated pathways in each transgenic zebrafish model and subgroups of human HCCs. Briefly, the entire transcriptome was ranked by logarithm transformed p-value (base 10). The up-regulated genes were given positive values, and the down-regulated genes negative values. The curated canonical pathways from the MSigDB (Molecular Signature Database) were used. The number of permutation used was 1000.

### RT-qPCR Validation

Total RNA were reverse-transcribed using the SuperScript II cDNA Synthesis Kit (Invitrogen). RT-qPCR was performed with same sets of cDNAs used for SAGE sequencing using the LightCycler 480 SYBR Green I Master system (Roche). Reactions were conducted in triplicate for each cDNA sample and primer sequences are shown in the Table S4. Gene expression levels in each control or transgenic liver sample were normalized with the level of *β-actin* mRNA as the internal control. The log_2_ fold changes between tumor and control samples were calculated using the CT method according to the formula: log_2_ fold changes = −ΔΔCT = −[(CT gene of interest–CT *β-actin*) transgenic sample–(CT gene of interest–CT β-actin) control sample]. Two-tailed heteroscedastic t test was performed using normalized CT values (CT gene - CT β-actin) and changes with p<0.05 are considered to be significant.

## Results

### Identification of Differentially Expressed Genes in the Three Transgenic Zebrafish Liver Cancer Models

**Figure 1:**
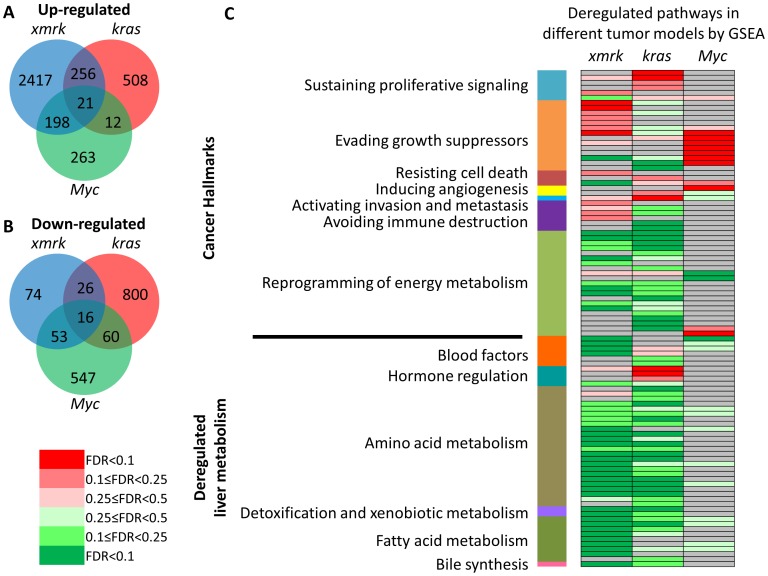
Deregulated genes and pathways in *xmrk*, *kras* and *Myc* transgenic liver tumors. (A) Venn diagram of up-regulated genes in the three zebrafish transgenic HCC models. (B) Venn diagram of down-regulated genes in the three zebrafish transgenic HCC models. (C) Deregulated pathways in the three oncogene transgenic liver tumors. Up- and down-regulated pathways in each zebrafish transgenic HCC model were analyzed by GSEA pre-ranked analysis. Red and green indicate up-and down-regulation respectively and the color code based on FDR is shown on the left. Pathways correlating with grey cells were not detected with any change. The up-regulated pathways were assigned into seven cancer hallmark categories (excluding enabling replicative immortality) according to Hanahan and Weinberg [27] and down-regulated pathways was classified based on different aspects of the liver metabolism (see Table S1 for details).

The three oncogene transgenic lines (*xmrk, Myc* and *kras^V12^*) were induced to develop liver tumors (Figure S1) and these tumor samples were subjected to RNA-SAGE sequencing. By a selection criteria of fold change>1.5, p value<0.05 and TPM>10 (in either control or tumor samples), differentially expressed genes were selected from the three tumor sets. There were 2,892, 797 and 494 genes up-regulated and 169, 902 and 676 genes down-regulated in the *xmrk*-, *kras*- and *Myc*- induced zebrafish liver cancer, respectively (Figure 1A,B). Deregulated genes from the three transgenic models showed relatively small overlaps, indicating that the three oncogenes regulated quite distinct sets of genes. This is consistent with the report that morphologically uniform cancer type is frequently classified into different subgroups based on their distinct gene expression patterns [26]. Interestingly, there were 21 up-regulated and 16 down-regulated genes commonly found in all the three tumor models (Figure 1A, B, Table 1).

**Table 1:** Commonly deregulated genes in the three transgenic zebrafish liver cancer models.

	Common	Gene Symbol	Human homolog Gene Symbol	*xmrk*		*kras*		*Myc*	
				FC	P value	FC	P value	FC	P value
Up	18858688	*foxa3*	*FOXA3*	3.58	6.66E-03	1.78	4.16E-02	1.55	9.18E-03
	18858728	*gart*	*GART*	3.32	1.42E-03	2.87	5.87E-03	7.69	6.93E-04
	18859502	*tp53*	*TP53*	5.09	1.43E-03	1.92	1.09E-02	3.61	3.30E-03
	41054118	*c20orf24*	*C20orf24*	4.92	2.13E-03	1.93	2.83E-03	3.14	5.22E-03
	41393080	*itm1*	*STT3A*	1.98	7.59E-03	1.56	3.02E-02	2.01	8.07E-04
	47086630	*rhot1a*	*RHOT1*	1.62	3.11E-02	1.87	3.00E-02	1.58	3.39E-02
	47087206	*abce1*	*ABCE1*	4.12	1.97E-03	6.79	1.25E-02	3.01	4.40E-03
	50344865	*cdkrap3*	*CDK5RAP3*	2.09	4.98E-03	1.85	2.06E-02	2.42	1.43E-03
	50345019	*mgat4b*	*MGAT4B*	4.09	1.62E-02	20.55	2.92E-02	1.70	3.03E-03
	50540209	*srprb*	*SRPRB*	1.85	2.00E-02	4.47	6.89E-04	2.65	5.84E-03
	55742596	*eif5a2*	*EIF5A2*	4.83	1.09E-03	1.45	4.07E-03	2.98	2.14E-04
	62955566	*cirbp*	*CIRBP*	6.11	1.63E-03	2.00	3.17E-02	2.16	1.44E-02
	66773145	*noc4l*	*NOC4L*	2.67	4.20E-02	1.63	3.58E-02	4.34	1.05E-02
	71834591	*reep2*	*REEP2*	4.38	3.51E-02	7.17	3.26E-03	8.38	7.95E-03
	76253887	*srp14*	*SRP14*	4.17	1.62E-03	1.71	1.61E-02	1.91	1.15E-04
	94536632	*stmn1a*	*STMN1*	13.83	2.30E-03	3.05	4.93E-03	4.30	1.56E-02
	113679133	*mrps9*	*MRPS9*	1.71	2.85E-02	1.56	3.71E-02	2.40	1.59E-02
	121583749	*hmgcra*	*HMGCR*	7.42	5.42E-03	30.45	7.30E-04	8.71	2.41E-03
	126723627	*ubap2*	*UBAP2*	1.94	3.33E-02	1.88	4.99E-03	1.58	9.64E-03
	148225559	*fam162a*	*FAM162A*	2.15	2.76E-03	2.16	4.72E-02	1.97	2.22E-02
	169646807	*rrp9*	*RRP9*	4.20	1.01E-02	2.99	3.32E-02	2.71	3.48E-02
Down	23308680	*cyp2ad2*	*–*	4.35	1.99E-02	9.09	8.07E-03	2.94	2.73E-02
	41053663	*scp2*	*SCP2*	5.00	9.46E-03	2.94	2.02E-02	1.96	2.17E-02
	41055025	*hsd17b3*	*HSD17B3*	10.00	3.20E-02	100.00	7.61E-03	33.33	1.66E-02
	41152446	*hsdl2*	*HSDL2*	3.85	4.21E-02	2.13	3.55E-02	2.56	6.38E-03
	47085884	*fbp1b*	*FBP1*	9.09	3.91E-04	8.33	1.86E-02	2.38	2.87E-02
	47086066	*tdh*	*TDH*	6.67	3.28E-02	2.86	1.50E-02	3.45	3.93E-02
	47086178	*itgb1b.2*	*ITGB1*	7.14	4.19E-02	7.14	6.40E-03	4.35	3.30E-03
	47086928	*ak3l1*	*AK3L1*	2.17	2.31E-02	10.00	1.56E-03	2.00	2.17E-02
	54400637	*ech1*	*ECH1*	1.52	2.29E-02	2.17	3.78E-03	2.00	1.51E-02
	55925455	*gpx4a*	*GPX4*	3.57	6.79E-03	2.27	3.32E-02	5.26	7.31E-03
	56790261	*sod1*	*SOD1*	2.94	4.18E-02	1.85	5.32E-03	2.27	2.48E-02
	70778900	*slc27a2*	*SLC27A2*	9.09	3.26E-02	5.00	5.13E-03	2.78	1.08E-02
	71834285	*apobl*	*APOB*	5.26	4.21E-02	1.79	1.20E-02	1.75	1.46E-02
	71834671	*miox*	*MIOX*	3.13	3.05E-02	9.09	8.07E-03	4.55	4.30E-02
	121583789	*nrxn1b*	*NRXN1*	4.76	4.87E-03	1.96	2.18E-02	6.67	1.51E-02
	148230211	*slco1d1*	*–*	3.70	6.22E-03	4.17	1.00E-04	2.70	1.11E-03

### Distinct Pathways Regulated in the Three Zebrafish Liver Tumor Models

Pathway analysis using GSEA showed that the three transgenic liver cancer models have different pathways deregulated (Figure 1C, Table S5). It has been widely accepted that there are eight cancer hallmarks for multistep tumorigenesis and the complexities of neoplasms [27], [28]. We found that the GSEA-identified pathways fallen into at least seven cancer hallmarks (except for enabling replicative immortality). *Xmrk* mainly up-regulated pathways involved in evading growth suppressors and avoiding immune destruction, which included activating cell cycle, promoting RNA transcription, up-regulating proteasome and altering immune properties. *Kras* provided the tumor cells with the ability of self-sustaining proliferative signals by up-regulating EGFR, Raf-MEK-ERK, PI3K-AKT-mTOR and GSK3 signaling pathways. Specifically, it also altered the focal adhesive characters of tumor cells which could activate invasion and metastasis. *Myc* mainly up-regulated translation and proteolysis to assist tumor cells to evade growth suppressors, and it also up-regulated VEGF pathway, thus potentially inducing angiogenesis. While there was no single pathway significantly up-regulated in all three tumor models, there were some pathways up-regulated in two models, such as proteasome in the *xmrk* and *Myc* models, and IGF1 pathway, mTOR pathway, tRNA biosynthesis and focal adhesion in the *xmrk* and *kras* models. In contrast, pathways in reprogramming of energy metabolism were generally down-regulated in all three models though the down-regulation in the *Myc* model was less apparent than the other two models. Meanwhile, many other pathways involved in normal liver function such as blood factors, amino acid metabolism, detoxification and xenobiotic metabolism, fatty acid metabolism, and bile synthesis were uniformly down-regulated in all three tumor models. However, one exception was hormone regulation that was apparently up-regulated in the *kras* tumors.

### Correlation of the Three Gene Signatures of Zebrafish Liver Tumors with Different Stages of Human HCCs

**Figure 2:**
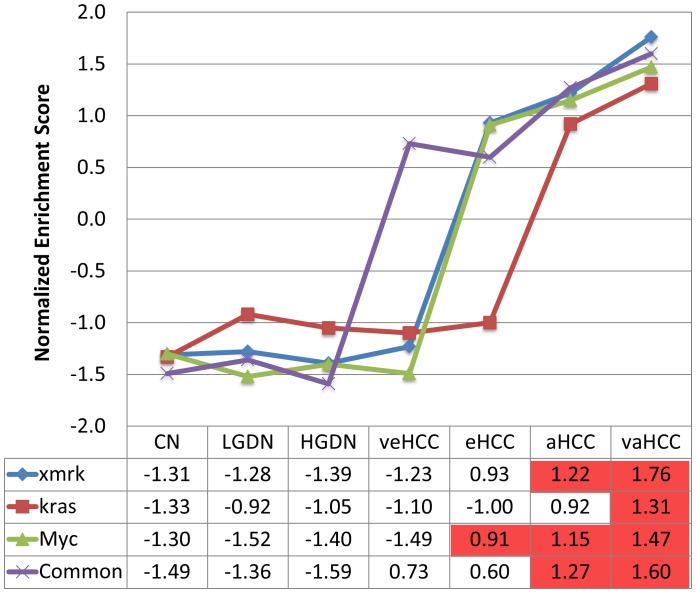
Correlation of the zebrafish liver tumor signatures with different stages of human hepatocarcinogenesis. The up-regulated gene signatures from the three transgenic tumors (xmrk, kras and Myc) and 21 commonly up-regulated genes (Common) were used for GSEA and NESs (normalized enrichment scores (y-axis) are plotted against different stages of human HCCs (x-axis). NES is listed in the table and asterisk indicate statistical significance: FDR<0.25. Abbreviations: CL, cirrhotic livers; LGDN, low grade dysplastic nodules; HGDN, high grade dysplastic nodules; veHCC, very early HCC; eHCC, early HCC; aHCC, advanced HCC; vaHCC, very advanced HCC.

The up-regulated genes, 2,892, 797 and 494 from *xmrk*, *kras* and *Myc* tumors respectively, were used as the signature genes for each model. These up-regulated genes were converted to 1,362, 490, and 146 human Unigenes respectively in order to compare with available transcriptomic data from human studies. We then compared the three signature gene sets with a set of human transcriptomic data (GSE6764) from different stages of human liver conditions: cirrhotic liver, low grade dysplastic nodules (LGDN), high grade dysplastic nodules (HGDN), and very early, early, advanced, and very advanced HCC (veHCC, eHCC, aHCC and vaHCC), in which the pathological HCC stages have been defined by tumor size, differentiation status and metastasis level [25]. The three signature gene sets were all up-regulated as the disease progresses, but they started to be up-regulated at different stages of tumorigenesis (Figure 2). The *xmrk* signature showed positive correlation with HCCs starting from eHCC, and it was significantly correlated with aHCC and vaHCC. The *kras* signature positively correlated with aHCC and vaHCC, and it was only significantly correlated with vaHCC. The *Myc* signature showed significantly positive correlation with HCCs starting from eHCC, similar with our previous result using an independent set of RNA-seq data from the same transgenic line [6]. Interestingly, the common 21 up-regulated genes in all three tumor models also showed up-regulation even from the very early stage of HCC, indicating these genes are correlated with the entire neoplastic process.

### Representation of the Three Gene Signatures of Zebrafish Liver Tumors in Human HCC Samples

Given the distinctive pathways altered in the three zebrafish liver tumor models and the high level of heterogeneity in human HCC patients, we sought to examine the degree of representation of the three zebrafish liver cancer models in human HCC samples. We examined ten sets of published clinical HCC microarray data, each of which included at least 80 clinical samples. The ten datasets contain a total of 1,272 samples covering different ethnic groups and risk factors (Table S2). We found that the *xmrk*, *kras* and *Myc* gene signatures were significantly correlated with 30.8%, 24.8% and 25.6% of all clinical samples, respectively. 47.2% of the human HCC samples had significant correlation with at least one of the three zebrafish gene signatures (Table 2, Figure S2). In different human HCC datasets, the percentage ranges from 22.0% to 60.4%. Moreover, some of the clinical samples were correlated with two or even three zebrafish gene signatures. Co-correlation of two gene signatures, namely *xmrk*/*kras*, *kras*/*Myc* and *kras*/*Myc,* accounted for 17.5%, 13.5% and 12.4% of the human HCC samples. 9.3% of the human samples showed co-correlation of all the three gene signatures. Thus, it appears that the three transgenic zebrafish liver tumor models represent molecular mechanisms of hepatocarcinogenesis in almost half of the human HCC cases and the other half of human HCC may be due to different molecular mechanisms.

**Table 2:** Degree of representation of the three transgenic zebrafish liver cancer signatures in human HCCs.

	GEO accession	*xmrk*	*kras*	*Myc*	Total*	*xmrk*/*kras*	*xmrk*/*Myc*	*kras*/*Myc*	*xmrk*/*kras*/*Myc*
A	GSE364	12.6%	17.2%	19.5%	31.0%	8.0%	6.9%	6.9%	3.4%
B	GSE1898	9.9%	11.0%	16.5%	22.0%	8.8%	7.7%	7.7%	6.6%
C	GSE10141	28.8%	25.0%	17.5%	46.3%	16.3%	7.5%	5.0%	3.8%
D	GSE9843	35.2%	23.1%	25.3%	54.9%	14.3%	9.9%	8.8%	4.4%
E	GSE19977	37.8%	21.3%	25.0%	45.1%	20.1%	18.9%	13.4%	13.4%
F	GSE10186	32.2%	27.1%	12.7%	44.9%	19.5%	5.1%	6.8%	4.2%
G	GSE20017	44.4%	23.7%	30.4%	50.4%	23.0%	24.4%	17.8%	17.0%
H	GSE25097	34.7%	31.0%	34.7%	60.4%	20.1%	14.9%	15.3%	10.4%
I	GSE5975	26.9%	32.4%	32.4%	46.2%	22.3%	17.2%	21.4%	15.5%
J	GSE14520	30.5%	20.0%	20.5%	47.6%	11.4%	10.0%	6.2%	3.3%
	Total	29.2%	23.5%	23.8%	47.2%	16.9%	12.5%	11.5%	8.8%

Percentages indicate the percentages of human HCC samples which showed significant positive correlation (FDR<0.25) with the zebrafish liver cancer signatures.

*Total: The total percentage of human HCCs which showed significant correlation with any one or more of the zebrafish signatures.

**Figure 3:**
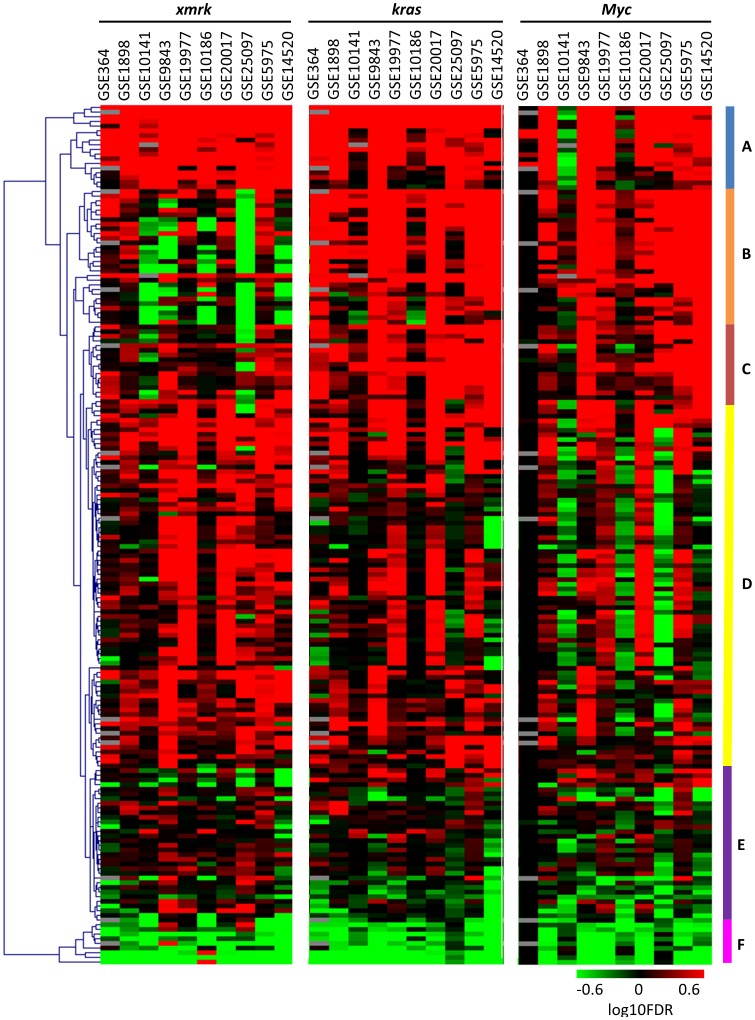
Hierarchical clustering of pathways differentially expressed in the subgroups of human HCCs significantly correlated with the three zebrafish tumor signatures. The color bars represent the logarithm-transformed FDR values. Up-regulated pathways are given positive values (red) and down-regulated pathways negative values (green). Pathways correlating with grey cells are not detected in the pathway analyses. Pathways with either FDR = 1 or not detected in more than five out of the 30 combinations are pre-excluded from the analysis.

We further demonstrated that the subsets of human HCCs which showed significantly positive correlation with the same gene signature also shared similar up-regulated pathways (Figure 3, Table S6). For this analysis, each of the human HCC sets was separated into two subgroups: those showing significant correlation with one of the zebrafish signatures, and the rest. Differentially expressed genes between the two groups were identified by two-tailed t-test, and pathway analyses were performed by GSEA pre-ranked analysis. The pathways were subjected to hierarchical clustering by logarithm-transformed FDR values using MeV [29], [30]. As shown in Figure 3, the pathways differentially regulated in human HCCs which showed significant correlation with one of the zebrafish signatures had distinct patterns. The pathway cluster A is consisted of pathways up-regulated in all the three subgroups, including proteasome, tRNA biosynthesis and oxidative phosphorylation. Ribosome, transcription and translation, and cell cycle were also highly up-regulated, suggesting that the human HCC samples significantly correlated with any of the zebrafish signatures were probably more proliferative than those not significantly correlated. The pathway cluster B contains pathways down-regulated in most of the subgroups associated with *xmrk* but up-regulated in the subgroups associated with *kras* and *Myc*. Interestingly, theses pathways were consistent with highly down-regulated pathways in the *xmrk*-induced zebrafish HCC, including energy metabolism, amino acid metabolism, fatty acid metabolism, bile acid biosynthesis, complement pathway, biosynthesis of steroid, and N-glycan biosynthesis. The pathway cluster C is more up-regulated in the *kras*-associated HCC subgroups, including glutamate metabolism and glycolysis. It has been reported that *Kras* could increase the conversion of glucose to glutamate, and this was essential for *Kras*-mediated tumorigenicity [31]. The pathway cluster D was generally up-regulated in the *xmrk*- and *kras*-correlated human HCCs, but showed a disparate pattern in the *Myc*-correlated human HCCs. This cluster contained many kinase pathways which were deregulated in the *xmrk*- and *kras*-induced zebrafish liver cancer, but not significantly changed in the *Myc*-induced zebrafish liver cancer. The pathway cluster E was quite heterogeneous. The pathway cluster F was well separated from all the others and it contained pathways which were mostly down-regulated in all the human HCC subgroups associated with zebrafish signatures, including hematopoietic cell lineage, cytokine pathway, calcium signaling, and GPCR pathway. Since most of these down-regulated pathways are involved in inflammatory response, it is likely that the subgroups of human HCCs not captured by the three zebrafish models have more severe inflammatory status.

### The Commonly Up- and Down-regulated Genes in Zebrafish Liver Tumors are also Consistently Up- and Down-regulated in Human HCCs

**Figure 4:**
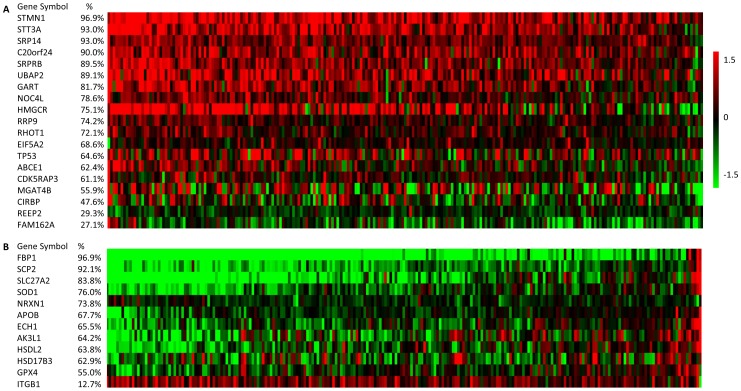
*In silico* validation of the 21 commonly up-regulated and 16 commonly down-regulated genes in human HCC dataset. 19 of the up-regulated genes and 12 of the down-regulated genes were identified in the microarray platform. The red color indicates up-regulation, and the green color indicates down-regulation.

While the three gene signatures represent up-regulation of distinct pathways and correlate with different subgroups of human HCCs, we sought to investigate whether the 21 commonly up-regulated and 16 commonly down-regulated genes in all three transgenic models would be similarly regulated in human HCCs. Among the ten human HCC datasets we used, we were able to examine two datasets which included both HCCs and their corresponding non-tumor tissues (GSE14520 and GSE25097). However, only GSE14520 could be compared with the zebrafish data as most of the human genes homologous to the common zebrafish genes could be identified from the microarray platform used: 19 of the 21 up-regulated genes (except *FOXA3* and *MRPS9*) and 12 of the 16 down-regulated genes (except for *cyp2ad2*, *slco1d1*, *TDH* and *MIOX)*. GSE14520 dataset contains 229 primary HCCs and the corresponding paired non-tumor hepatic tissues [32], [33]. The *xmrk*, *kras* and *Myc* signatures were significantly correlated with 30.5%, 20.0%, and 20.5% of the HCC samples, and in total 47.6% of the HCC samples showed significant up-regulation of at least one of the zebrafish liver cancer signatures (Table 2). Among the 19 up-regulated genes, 16 of them were up-regulated in more than half of the HCC patients, and 9 of them were up-regulated in more than 75% of the patients (Figure 4). *STMN1* was up-regulated in 96.9% of the human HCCs from the dataset, which is the most ubiquitously up-regulated. *STT3A* and *SRP14* were up-regulated in 93.0% of the human HCCs. Among the 12 down-regulated genes, 11 of them were down-regulated in more than half of the HCC patients. *FBP1* was down-regulated in 96.9% of the examined human HCCs. FBP1 is a gluconeogenesis regulatory enzyme and it functions to antagonize glycolysis in gastric cancer [34]. *FBP1* is down-regulated in majority of human HCCs by methylation [35]. Restoration of FBP1 expression in human HCC cell lines significantly inhibited cell growth, suggesting that it might function as a tumor suppressor [35].

Finally, the expression of these 19 up- and 16 down-regulated genes was also validated by RT-qPCR in all three different types of tumors. As shown in Figure S3, the majority of gene in the majority of tests (∼90%) confirmed consistent trend of changes in the tumor samples.

## Discussion

It is well known that human HCCs are highly heterogeneous; thus, cross-species comparative studies at the transcriptomic level should be valuable to identify conserved and critical pathways in carcinogenesis in vertebrate species. Here we first determined deregulated pathways from each of the oncogene transgenic zebrafish model. Although the three transgenic tumor models had quite distinct deregulated biological pathways, they all correlated to advanced or very advanced HCCs by comparison with gene signatures from human HCCs. Furthermore, we also found that each of the zebrafish model represent a subset of human HCCs. Since our oncogene transgenic lines have well-defined driving pathways in carcinogenesis, the information from the transgenic zebrafish should be valuable for understanding the main molecular mechanisms of each HCC subgroup, which is imperative for developing more effective therapeutics specific for each subgroup. Interestingly, our three oncogene transgenic zebrafish models significantly represent only less than half of the human HCCs and there is a need to develop more and different oncogene transgenic animal models for covering more human HCCs for further understanding of distinct molecular mechanisms in hepatocarcinogenesis, with focus on inflammatory pathways.

In the present study, we also identified a list of commonly deregulated genes in liver tumors induced by different oncogenic signals was identified and their expressional changes in human HCC samples were validated *in silico* (Table 1, Figure 4). These genes could be served as potential therapeutic targets since they were independent of individual oncogenic pathways. The up-regulated genes includes those in protein translation and processing (*eif5a2*, *abce1*, *rrp9*, *srp14*, *itm1*, *srprb*), pro-apoptosis (*rhot1a*, *c20orf24*, *fam162a*) anti-apoptosis (*tp53*), cell cycle regulation (*stmn1a*, *cdkrap3*), purine synthesis (*gart*), rRNA processing (*noc4l*), G protein-coupled receptors signaling (*reep2*), n-glycan biosynthesis (*mgat4b*), stress response (*cirbp*), ubiquitination (*ubap2*), peroxisome (*hmgcra*), mitochondrial function (*mrps9*) and transcription (*foxa3*). Some of them have been implicated in hepatocarcinogenesis or identified as therapeutic targets. For example, *hmgcra*, a top up-regulated gene in all three tumor models, encodes the rate-limiting enzyme for cholesterol synthesis. Inhibition of Hmgcr could block tumor cell growth and metastasis [36], but clinical trials with Hmgcr inhibitor (pravastatin) have shown discrepant results [37]–[39], which may be attributed to the genetic heterogeneity of HCCs. The fact that *hmgcra* was highly up-regulated in all of the three transgenic zebrafish liver tumor models and these tumor models represent about half of human HCCs may suggest that it should be a therapeutic target in a broad, though not all, range of HCCs. Another top up-regulated gene, *mgat4b,* is one of the important enzymes in the biosynthetic pathway of N-glycans. N-glycan is up-regulated in human HCC [40] and is associated with drug resistance [41]. Another gene which was highly up-regulated is *stmn1a*. The human homolog of *stmn1a*, *STATHMIN1,* is over-expressed and is associated with polyploidy, metastasis, early recurrence, and poor prognosis in hepatocarcinogenesis [42]–[44]. It has also been identified as a major molecular target of an anticancer drug [45].

Most of the 16 genes commonly down-regulated (Table 1) are apparently involved in metabolism for normal liver function, including fatty acid metabolism (*cyp2ad2*, *hsdl2*, *ech1*), intracellular fatty acid and lipid transport (*scp2*, *slc27a2*, *apobl*), androgen metabolism (*hsd17b3*), glucose metabolism (*fbp1b*), mitochondria function (*tdh*, *ak4*), integrin complex (*itgb1b.2*), antioxidation (*gpx4a*, *sod1*), inositol catabolism (*miox*), bile acid metabolism (*slco1d1*) and neuron cell adhesion (*nrxn1b*). Several of them may have a direct connection with hepatocarcinogenesis. For example, *Sod1* deficient mice showed extensive cellular oxidative damage and majority of them developed HCC [46]. Moreover, SOD1 has also been markedly down-regulated in human HCCs induced from different etiological factors [47]. Gpx4 has also been reported to be associated with multiple types of cancers, including breast cancer [48], colorectal cancer [49], and aggressive prostate cancer [50]; however, no study has presented its correlation with HCC. Both Sod1 and Gpx4 are important components of the cellular antioxidant mechanisms. The down-regulation of these two genes in the three transgenic zebrafish liver cancer models suggested that oxidative damage is a common and conserved part of hepatocarcinogenesis.

## Supporting Information

Figure S1 **Induction of liver tumors in the three zebrafish transgenic liver cancer models.** (A–D) Gross morphology of treated transgenic fish and the non-transgenic siblings. The treated non-transgenic siblings have normal liver size and gross morphology (A). The livers in the treated transgenic fish were obviously enlarged (B–D) compared to the treated non-transgenic siblings (A). (E–H) Histological examination of the treated transgenic fish and non-transgenic siblings. Treated *xmrk* fish developed HCC (F), treated *Myc* fish developed HCA (G), and treated *kras* fish developed heterogeneous HCC (H). The liver was circled out in white dotted lines. Scale bars: 25 mm for A–D, 50 µm for E–H.
(TIF)

Figure S2 **Correlation of the three transgenic zebrafish liver cancer signatures with human HCC samples.** The heat maps showed the positive- and negative-correlation of the three transgenic zebrafish liver cancer signatures with 9 sets of human HCC samples, including GSE364 (A), GSE1898 (B), GSE10141 (C), GSE9843 (D), GSE19977 (E), GSE10186 (F), GSE20017 (G), GSE25097 (H) and GSE5975 (I). The color was determined by normalized enrichment score (NES) of the GSEA analysis. The red color indicates up-regulation or positive correlation, and the green color indicates down-regulation or negative correlation.
(TIF)

Figure S3 **RT-qPCR validation of commonly up- and down-regulated genes in three transgenic zebrafish liver tumors.** (A) Up-regulated genes. (B) Down-regulated genes. Gene names are indicated at the top and transgenic lines are indicaed on the left. RT-qPCR data are presented with red bars in comparison with corresponding RNA-Seq data represented by blue bars. Y-axes indicate fold changes on Log2 scale. Standard error bars are included for the RT-qPCR data and asterisks indicate statistically significance (P<0.05).
(TIF)

Table S1 **Summary of RNA-SAGE data.**
(DOCX)

Table S2 **Significantary up-regulated zebrafish genes with mapped human homologs.**
(XLSX)

Table S3 **Summary of human HCC datasets used in the present Study.**
(DOCX)

Table S4 **Sequences of PCR primers used for RT-qPCR validation of commonly up- and down-regulated genes in zebrafish liver tumors.**
(DOCX)

Table S5 **Details of pathways deregulated in the three transgenic zebrafish liver cancer models as classified into the seven cancer hallmarks and different aspects of the liver metabolisms.**
(DOCX)

Table S6 **Details of pathways differentially expressed in the subgroups of human HCCs which showed significantly correlation with the three zebrafish signatures.**
(DOCX)
