# Supplementary figures and images for: Xmrk, Kras and Myc Transgenic Zebrafish Liver Cancer Models Share Molecular Signatures with Subsets of Human Hepatocellular Carcinoma
Source: PLoS One. 2014 Mar 14;9(3):e91179. doi: 10.1371/journal.pone.0091179 (PMC3954698; doi:10.1371/journal.pone.0091179)

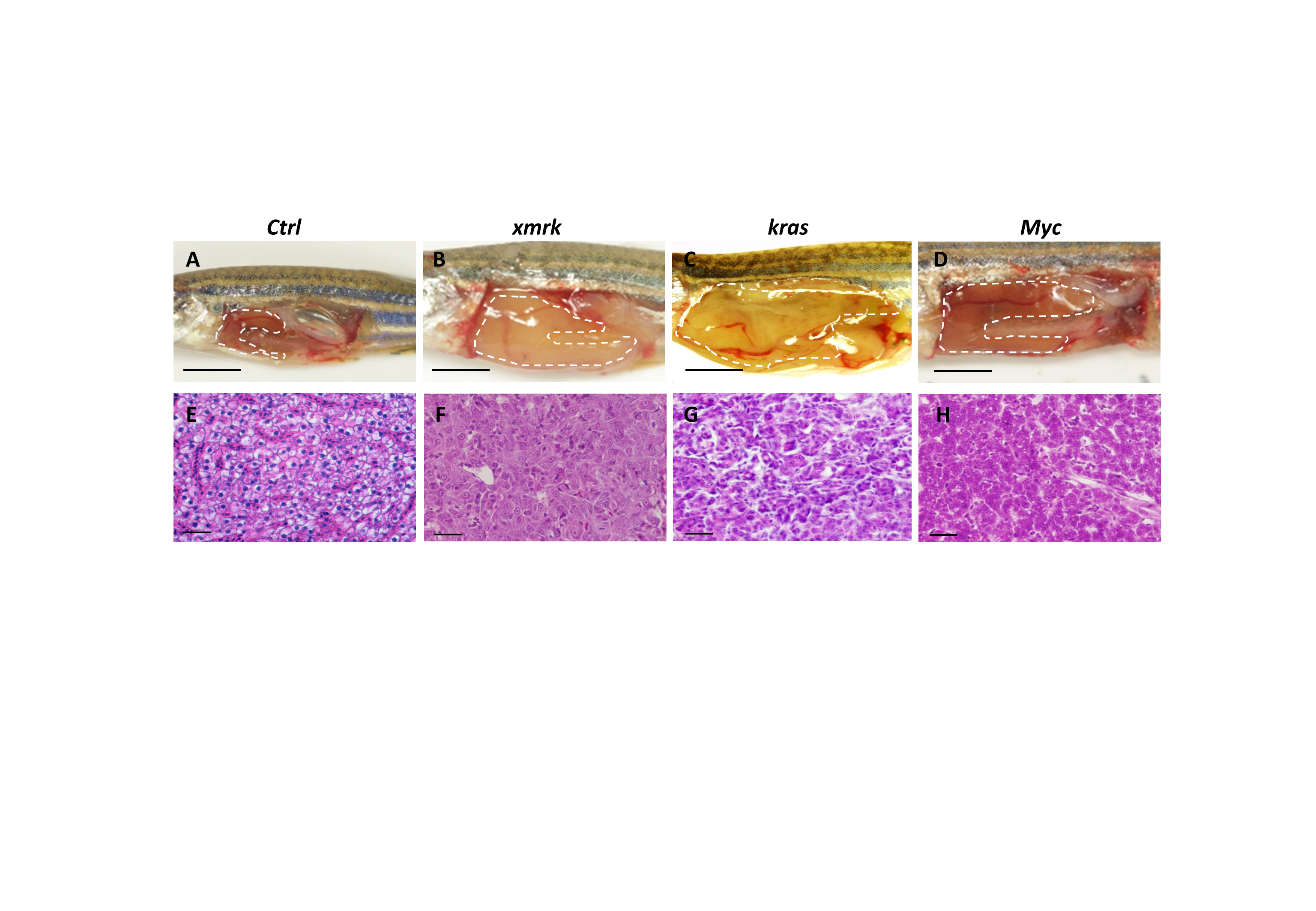

Supplement: Figure S1 — Induction of liver tumors in the three zebrafish transgenic liver cancer models. (A–D) Gross morphology of treated transgenic fish and the non-transgenic siblings. The treated non-transgenic siblings have normal liver size and gross morphology (A). The livers in the treated transgenic fish were obviously enlarged (B–D) compared to the treated non-transgenic siblings (A). (E–H) Histological examination of the treated transgenic fish and non-transgenic siblings. Treated xmrk fish developed HCC (F), treated Myc fish developed HCA (G), and treated kras fish developed heterogeneous HCC (H). The liver was circled out in white dotted lines. Scale bars: 25 mm for A–D, 50 µm for E–H. (TIF) [file pone.0091179.s001.tif]

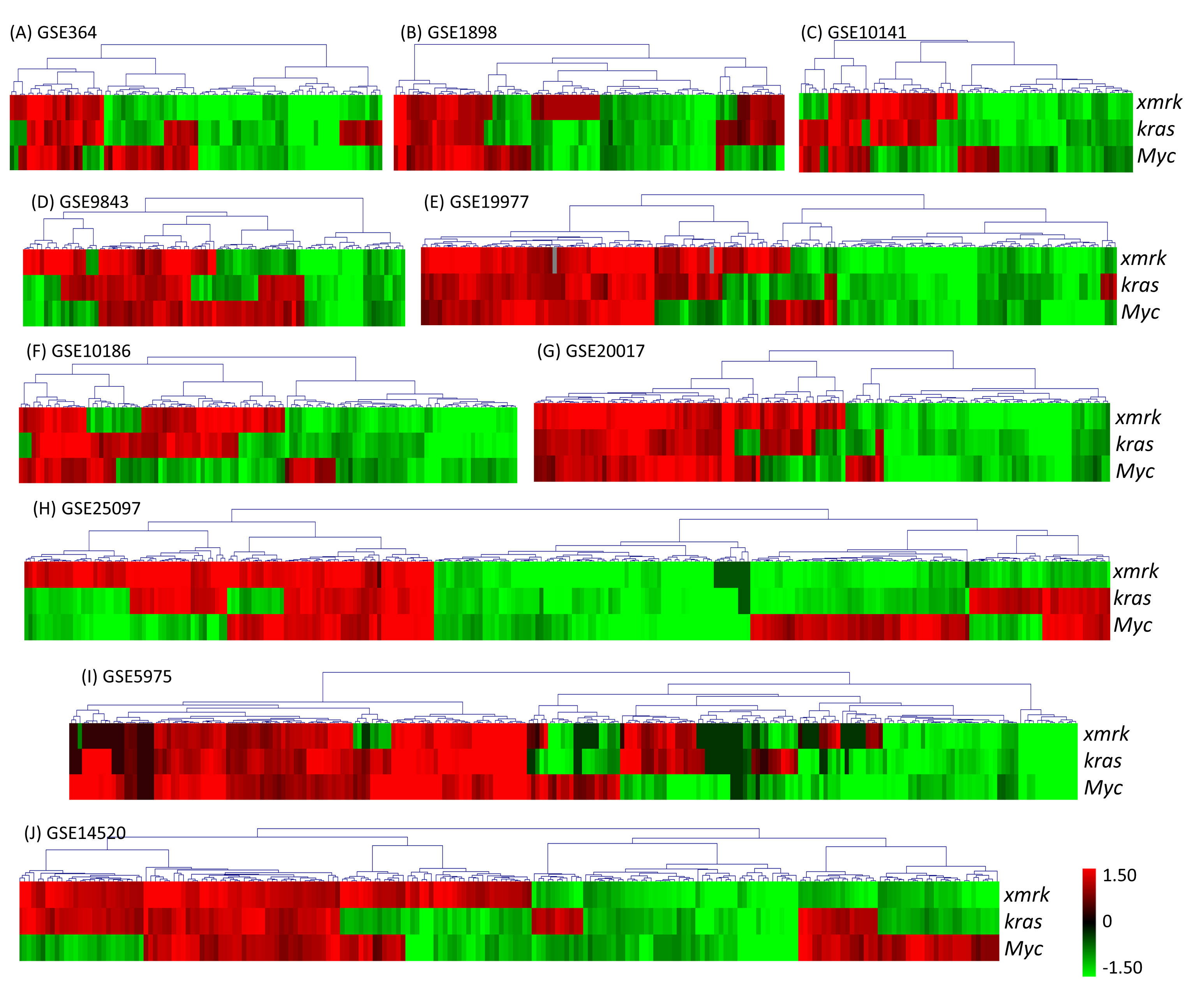

Supplement: Figure S2 — Correlation of the three transgenic zebrafish liver cancer signatures with human HCC samples. The heat maps showed the positive- and negative-correlation of the three transgenic zebrafish liver cancer signatures with 9 sets of human HCC samples, including GSE364 (A), GSE1898 (B), GSE10141 (C), GSE9843 (D), GSE19977 (E), GSE10186 (F), GSE20017 (G), GSE25097 (H) and GSE5975 (I). The color was determined by normalized enrichment score (NES) of the GSEA analysis. The red color indicates up-regulation or positive correlation, and the green color indicates down-regulation or negative correlation. (TIF) [file pone.0091179.s002.tif]

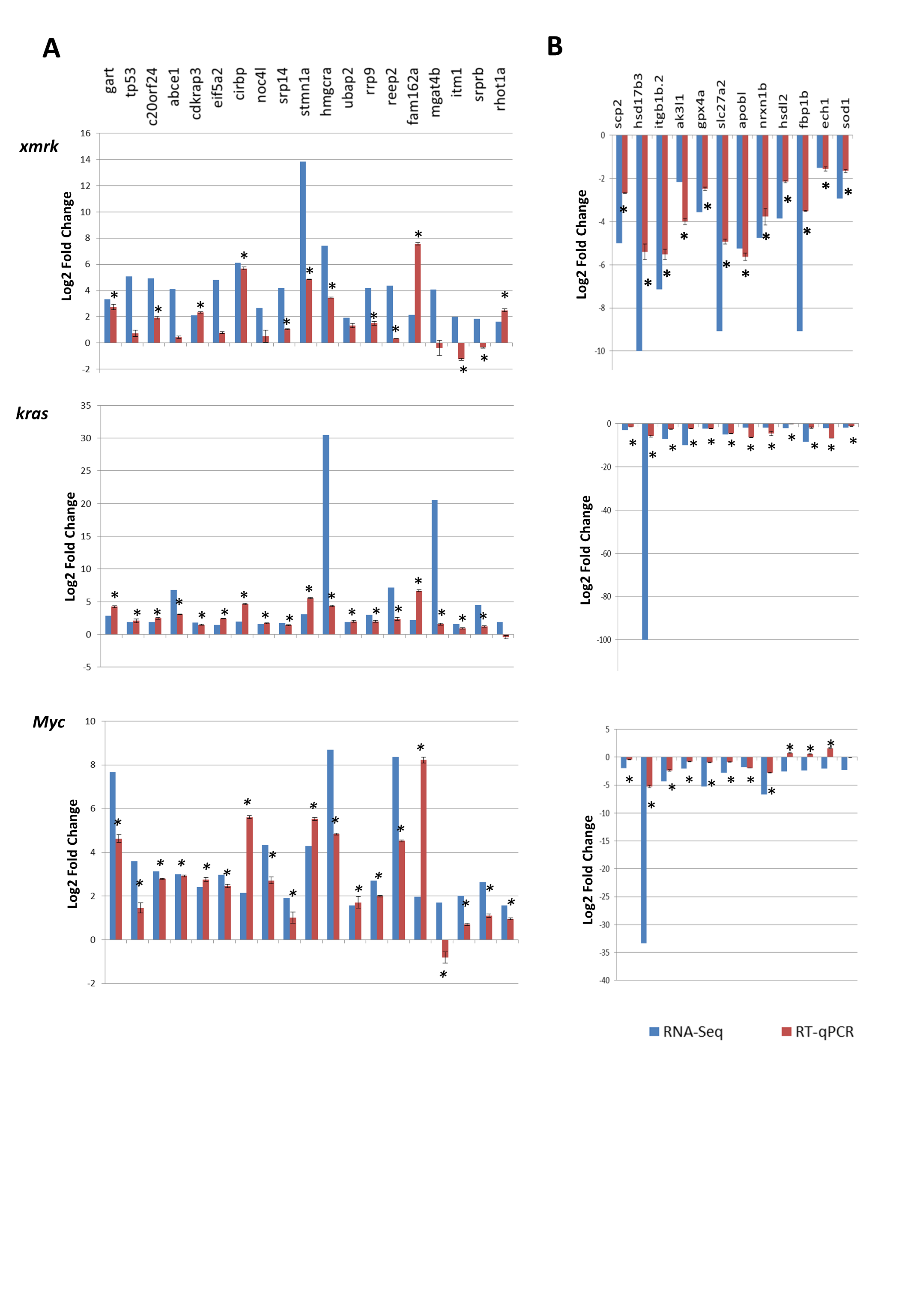

Supplement: Figure S3 — RT-qPCR validation of commonly up- and down-regulated genes in three transgenic zebrafish liver tumors. (A) Up-regulated genes. (B) Down-regulated genes. Gene names are indicated at the top and transgenic lines are indicaed on the left. RT-qPCR data are presented with red bars in comparison with corresponding RNA-Seq data represented by blue bars. Y-axes indicate fold changes on Log2 scale. Standard error bars are included for the RT-qPCR data and asterisks indicate statistically significance (P<0.05). (TIF) [file pone.0091179.s003.tif]
