# Supplementary material for: Xmrk, Kras and Myc Transgenic Zebrafish Liver Cancer Models Share Molecular Signatures with Subsets of Human Hepatocellular Carcinoma
Source: PLoS One. 2014 Mar 14;9(3):e91179. doi: 10.1371/journal.pone.0091179 (PMC3954698; doi:10.1371/journal.pone.0091179)
Supplement: Table S1 — Summary of RNA-SAGE data. (DOCX) [file pone.0091179.s004.docx]

**Table S1. Summary of RNA-SAGE data**

|  | Total tag reads | RefSeq entries |
| --- | --- | --- |
| X-M-D- | 14,937,923 | 13,956 |
| X+M-D- | 18,129,256 | 12,478 |
| X-M+D- | 11,929,959 | 13,592 |
| X-M-D+ | 14,390,779 | 13,465 |
| X+M-D+ | 10,500,931 | 13,277 |
| X-M+D+ | 14,998,311 | 12,494 |
| KC1 | 22,319,162 | 12,159 |
| KC2 | 20,809,613 | 11,757 |
| KT1 | 21,749,743 | 12,691 |
| KT2 | 23,397,434 | 11,181 |

Abbreviations: X, *xmrk*; M, *Myc*; D, doxycycline treatment; KC, *kras* transgenic fish without mifepristone induction; KT, *kras* transgenic fish with mifepristone induction.
