# Supplementary material for: Xmrk, Kras and Myc Transgenic Zebrafish Liver Cancer Models Share Molecular Signatures with Subsets of Human Hepatocellular Carcinoma
Source: PLoS One. 2014 Mar 14;9(3):e91179. doi: 10.1371/journal.pone.0091179 (PMC3954698; doi:10.1371/journal.pone.0091179)
Supplement: Table S3 — Summary of human HCC datasets used in the present Study. (DOCX) [file pone.0091179.s006.docx]

**Table S3. Summary of human HCC datasets used in the present study**

|  | GEO accession | Origin of samples | Patient etiology | Number of samples | Technical platform | References |
| --- | --- | --- | --- | --- | --- | --- |
| A | GSE364 | China | 97.5% male, 97.5% HBV | 87 | GPL257 NCI_UniGEM2_HCC | (1) |
| B | GSE1898 | China, Belgium | 78.9% male, 62.2% HBV and 12.2% HCV | 91 | GPL1528 NCI/ATC Hs-OperonV2 | (2, 3) |
| C | GSE10141 | Japan |  | 80 | GPL5474 Illumina Human 6k Transcriptionally Informative Gene Panel for DASL | (4, 5) |
| D | GSE9843 | Spain, Italy, USA | 66.7% male, 100% HCV | 91 | GPL570 Affymetrix Human Genome U133 Plus 2.0 Array | (5-7) |
| E | GSE19977 | USA, Italy, Spain, Japan | 73.3% male, 23.1% HBV and 61.5% HCV | 164 | GPL8432 Illumina HumanRef-8 WG-DASL v3.0 | (5, 7) |
| F | GSE10186 | Japan | 81.4% male, 23.7 HBV and 69.5% HCV | 118 | GPL5474 Illumina Human 6k Transcriptionally Informative Gene Panel for DASL | (8) |
| G | GSE20017 | Spain, Italy, USA | 75.6% male, 33.3% HBV and 40.0% HCV | 135 | GPL8432 Illumina HumanRef-8 WG-DASL v3.0 | (9) |
| H | GSE25097 | China | 80.4% male, 86.1% HBV | 268 | GPL10687 Rosetta/Merck Human RSTA Affymetrix 1.0, custom CDF | (10, 11) |
| I | GSE5975 | China | 100%HBV | 238 | GPL1528 NCI/ATC Hs-OperonV2 | (12) |
| J | GSE14520 | China | Mainly HBV | 210 | GPL570 Affymetrix Human Genome U133 Plus 2.0 Array | (13, 14) |

**References**

1. Ye QH, Qin LX, Forgues M, He P, Kim JW, Peng AC, Simon R, et al. Predicting hepatitis B virus-positive metastatic hepatocellular carcinomas using gene expression profiling and supervised machine learning. Nat Med 2003;9:416-423.

2. Lee JS, Chu IS, Mikaelyan A, Calvisi DF, Heo J, Reddy JK, Thorgeirsson SS. Application of comparative functional genomics to identify best-fit mouse models to study human cancer. Nat Genet 2004;36:1306-1311.

3. Lee JS, Heo J, Libbrecht L, Chu IS, Kaposi-Novak P, Calvisi DF, Mikaelyan A, et al. A novel prognostic subtype of human hepatocellular carcinoma derived from hepatic progenitor cells. Nat Med 2006;12:410-416.

4. Hoshida Y, Villanueva A, Kobayashi M, Peix J, Chiang DY, Camargo A, Gupta S, et al. Gene expression in fixed tissues and outcome in hepatocellular carcinoma. N Engl J Med 2008;359:1995-2004.

5. Villanueva A, Hoshida Y, Battiston C, Tovar V, Sia D, Alsinet C, Cornella H, et al. Combining clinical, pathology, and gene expression data to predict recurrence of hepatocellular carcinoma. Gastroenterology 2011;140:1501-1512 e1502.

6. Chiang DY, Villanueva A, Hoshida Y, Peix J, Newell P, Minguez B, LeBlanc AC, et al. Focal gains of VEGFA and molecular classification of hepatocellular carcinoma. Cancer Res 2008;68:6779-6788.

7. Toffanin S, Hoshida Y, Lachenmayer A, Villanueva A, Cabellos L, Minguez B, Savic R, et al. MicroRNA-based classification of hepatocellular carcinoma and oncogenic role of miR-517a. Gastroenterology 2011;140:1618-1628 e1616.

8. Hoshida Y, Nijman SM, Kobayashi M, Chan JA, Brunet JP, Chiang DY, Villanueva A, et al. Integrative transcriptome analysis reveals common molecular subclasses of human hepatocellular carcinoma. Cancer Res 2009;69:7385-7392.

9. Minguez B, Hoshida Y, Villanueva A, Toffanin S, Cabellos L, Thung S, Mandeli J, et al. Gene-expression signature of vascular invasion in hepatocellular carcinoma. J Hepatol 2011;55:1325-1331.

10. Lamb JR, Zhang C, Xie T, Wang K, Zhang B, Hao K, Chudin E, et al. Predictive genes in adjacent normal tissue are preferentially altered by sCNV during tumorigenesis in liver cancer and may rate limiting. PLoS One 2011;6:e20090.

11. Tung EK, Mak CK, Fatima S, Lo RC, Zhao H, Zhang C, Dai H, et al. Clinicopathological and prognostic significance of serum and tissue Dickkopf-1 levels in human hepatocellular carcinoma. Liver Int 2011;31:1494-1504.

12. Jia HL, Ye QH, Qin LX, Budhu A, Forgues M, Chen Y, Liu YK, et al. Gene expression profiling reveals potential biomarkers of human hepatocellular carcinoma. Clin Cancer Res 2007;13:1133-1139.

13. Roessler S, Jia HL, Budhu A, Forgues M, Ye QH, Lee JS, Thorgeirsson SS, et al. A unique metastasis gene signature enables prediction of tumor relapse in early-stage hepatocellular carcinoma patients. Cancer Res 2010;70:10202-10212.

14. Roessler S, Long EL, Budhu A, Chen Y, Zhao X, Ji J, Walker R, et al. Integrative genomic identification of genes on 8p associated with hepatocellular carcinoma progression and patient survival. Gastroenterology 2012;142:957-966 e912.
