# Supplementary material for: Xmrk, Kras and Myc Transgenic Zebrafish Liver Cancer Models Share Molecular Signatures with Subsets of Human Hepatocellular Carcinoma
Source: PLoS One. 2014 Mar 14;9(3):e91179. doi: 10.1371/journal.pone.0091179 (PMC3954698; doi:10.1371/journal.pone.0091179)
Supplement: Table S4 — Sequences of PCR primers used for RT-qPCR validation of commonly up- and down-regulated genes in zebrafish liver tumors. (DOCX) [file pone.0091179.s007.docx]

**Table S4. Sequences of PCR primers used for RT-qPCR validation of commonly up- and down-regulated genes in zebrafish liver tumors**

|  | **gene** | **RefSeq** | **Forward primer 5'-3'** | **Reverse primer 5'-3'** |
| --- | --- | --- | --- | --- |
| Up-regulated | *abce1* | NM_213051.2 | GGAGATTTGATGCTCCCCCT | CCTTTCGGCTTAGGATGGCA |
|  | *c20orf24* | NM_199850.1 | CGAAGCGGAAAGAGGAGGTT | TGATGCAGAATATGGCGATGC |
|  | *cdk5rap3* | NM_001002105.1 | ATCACCACGCTATGTGGAGC | GCAGCCAGCAGATCAATTCG |
|  | *cirbp* | NM_001017797.1 | AGTCGCTAGAGGACGCCTT | TCCACAGACTTTCCGTTCATCCC |
|  | *eif5a2* | NM_213262.1 | CCTCCACCCACAACATGGAC | CAGCCAAAACAGAGACCAGAAAT |
|  | *fam162a* | NM_001089343.1 | TCGGCCGTCAGATTGGGATA | TCCTCTTTCTCTCTCTGGGCA |
|  | *gart* | NM_131617.1 | GCCGCTCTTCTTCCTGGATT | ACTGAAGCCGTTGCTGTGTA |
|  | *hmgcra* | NM_001079977.1 | CCGGAGCCTGTGATGAGTTT | GGGGAATTTGGGACTGAGCA |
|  | *itm1* | NM_201458.2 | TGGATCCATCGTACGCCAAG | ACATGAGACGCACCATCACA |
|  | *mgat4b* | NM_001002180.1 | TAAAGAGCGGGTCAGATGGC | CGATCAGCGGCAGGTCTAAT |
|  | *noc4l* | NM_001024397.1 | AATGGCTTGTTCGTCCCCAT | TACACTGGCAGATGCGTTGA |
|  | *reep2* | NM_001030227.1 | CCACGCTACCTCGTGCTAAA | CACTGTCTTGGTGGTCTGCT |
|  | *rhot1a* | NM_212704.1 | TGCGCTAAAGTCTACAAGAAACAC | AGGTCAGCTTGGGTCATGTG |
|  | *rrp9* | NM_001118895.1 | GTGCCTCAGGCTCCCATAAT | ATCGTCCCAATCTGTGCTCC |
|  | *srp14* | NM_001033741.1 | TCTGACAGAACTGACTCGGC | AGTGCTGACCACTGTGCTGAT |
|  | *srprb* | NM_001002572.1 | TGAGAGAGGCAACAGTTGGAC | CCAGGATGCTGTCTGTGAGG |
|  | *stmn1a* | NM_001040375.1 | TGGCTGCTACAAGTGACATTC | GCTCACGCTTTTCTGCTAGG |
|  | *tp53* | NM_131327.2 | ATTTAGGCTCAGGTTCCCG | GCCAAGTTATCTCCATCCG |
|  | *ubap2* | NM_001082838.1 | GGAGTGAAGATGTGAGTCTCTCTG | ACAGGGTGGCATGTGTGTAG |
|  |  |  |  |  |
| Down-regulated | *scp2* | NM_200865.1 | GTGAAAGATGGTCCCGGTG | TCATGGCCATCCCCATATTCC |
|  | *fbp1b* | NM_213132.1 | CCAGAAGATGGCAGTTCAC | TGGTGGATTGATTCAGGC |
|  | *slc27a2* | NM_001025299.1 | TTAGAGTCTGGTGCGTCCTG | ACGGTTCTGCTGCTTATGA |
|  | *sod1F* | NM_131294.1 | CCGTCTATTTCAATCAAGAGGGTGA | TTGCAACACCACTGGCATCA |
|  | *nrxn1b* | NM_001079959.1 | CAAACTGCACATCGTGAAGGG | CCTGGTGGGAATCGCTCAAT |
|  | *apobl* | NM_001030062.1 | TCGTGACATGAGCGAAGTGG | TGGTGTGGGAATGGTCAACT |
|  | *ech1* | NM_001006068.1 | CCAAAACCAGTGATTGTAGCTGTT | GGGAACACACGGCTGACCAA |
|  | *ak3l1* | NM_213299.1 | GATGACATCACAGGGGAGGC | CAGCGAGCATCTGACTGGAT |
|  | *hsdl2* | NM_199599.2 | GAGAAGTTTGGAGGGATTGAC | GCTGAGGTTTAGTATGTGAGGG |
|  | *hsd17b3* | NM_200364.1 | ACCCTGCAAGCTCCTTGAAA | CTCAACAAAAACCTTTGATGCTGC |
|  | *gpx4a* | NM_001007282.1 | AGGTTTACGCATCCTGGCTT | TCCTTCTCCACCACACTTGGA |
|  | *itgb1b.2* | NM_212928.1 | ACCTTCCATTCAGACGAACCC | CAATCACGTCCGTGCAGACA |
|  |  |  |  |  |
| Housekeeping gene | *bactin2* | NM_181601.3 | GAGAGAGGCTACAGCTTCAC | ACTCCTGCTTGCTAATCCAC |
|  |  |  |  |  |
