# Supplementary material for: Xmrk, Kras and Myc Transgenic Zebrafish Liver Cancer Models Share Molecular Signatures with Subsets of Human Hepatocellular Carcinoma
Source: PLoS One. 2014 Mar 14;9(3):e91179. doi: 10.1371/journal.pone.0091179 (PMC3954698; doi:10.1371/journal.pone.0091179)
Supplement: Table S5 — Details of pathways deregulated in the three transgenic zebrafish liver cancer models as classified into the seven cancer hallmarks and different aspects of the liver metabolisms. (DOCX) [file pone.0091179.s008.docx]

**Table S5. Details of pathways deregulated in the three transgenic zebrafish liver cancer models as classified into the seven cancer hallmarks and different aspects of the liver metabolisms**

|  |  | Pathway | *xmrk* | *kras* | *Myc* |
| --- | --- | --- | --- | --- | --- |
| Cancer hallmarks | Sustaining proliferative signaling | HSA04012_ERBB_SIGNALING_PATHWAY |  | 1.83 |  |
|  |  | HSA04010_MAPK_SIGNALING_PATHWAY | 1.2 | 2.09 |  |
|  |  | ST_PHOSPHOINOSITIDE_3_KINASE_PATHWAY |  | 1.63 |  |
|  |  | GSK3PATHWAY |  | 1.68 |  |
|  |  | UCALPAINPATHWAY | 1.69 |  |  |
|  |  | MTORPATHWAY | -1.32 | 1.31 | 1.52 |
|  | Evading growth suppressors | CELL_CYCLE_KEGG | 1.84 | -1.34 |  |
|  |  | G1_TO_S_CELL_CYCLE_REACTOME | 1.83 |  |  |
|  |  | DNA_REPLICATION_REACTOME | 1.63 | -1.26 |  |
|  |  | G2PATHWAY | 1.57 |  |  |
|  |  | RNA_TRANSCRIPTION_REACTOME | 1.14 |  |  |
|  |  | PROTEASOMEPATHWAY | 1.57 | -1.14 | 1.57 |
|  |  | HSA03050_PROTEASOME | 1.78 | -1.19 | 2.08 |
|  |  | HSA00970_AMINOACYL_TRNA_BIOSYNTHESIS |  | 1.53 | 2.3 |
|  |  | AMINOACYL_TRNA_BIOSYNTHESIS | 1.28 |  | 2.23 |
|  |  | TRANSLATION_FACTORS |  |  | 2.26 |
|  |  | EIF2PATHWAY |  |  | 1.8 |
|  |  | HSA03010_RIBOSOME | -2.15 | -1.2 | 3.82 |
|  |  | PURINE_METABOLISM |  | -1.66 | 1.82 |
|  |  | HSA00240_PYRIMIDINE_METABOLISM |  | -1.72 |  |
|  | Resisting cell death | ST_FAS_SIGNALING_PATHWAY | 1.56 |  |  |
|  |  | IGF1PATHWAY |  | 1.56 |  |
|  |  | IGF1MTORPATHWAY | -1.57 | 1.53 | 1.71 |
|  | Inducing angiogenesis | VEGFPATHWAY |  |  | 1.94 |
|  |  | HSA04370_VEGF_SIGNALING_PATHWAY |  | 1.63 | -1.46 |
|  | Activating invasion and metastasis | HSA04510_FOCAL_ADHESION | 1.41 | 1.94 | -1.49 |
|  | Reprogramming of energy metabolism | HSA00010_GLYCOLYSIS_AND_GLUCONEOGENESIS | -2.15 | -2.43 |  |
|  |  | GLYCOLYSIS_AND_GLUCONEOGENESIS | -2.23 | -2.12 |  |
|  |  | GLUCONEOGENESIS | -1.44 | -2.29 |  |
|  |  | GLYCOLYSIS | -1.39 | -2.3 |  |
|  |  | GLYCOLYSISPATHWAY |  | -1.54 |  |
|  |  | MITOCHONDRIAL_FATTY_ACID_BETAOXIDATION | -2.52 | -1.28 |  |
|  |  | HSA00062_FATTY_ACID_ELONGATION_IN_MITOCHONDRIA | -1.3 |  |  |
|  |  | MITOCHONDRIAPATHWAY |  | -1.53 |  |
|  |  | ATP_SYNTHESIS | 1.27 | 1.49 | -1.88 |
|  |  | HSA04920_ADIPOCYTOKINE_SIGNALING_PATHWAY |  |  | -2.02 |
|  |  | CITRATE_CYCLE_TCA_CYCLE | -1.33 | -1.63 |  |
|  |  | KREBS_TCA_CYCLE | -1.7 | -1.61 |  |
|  |  | HSA00020_CITRATE_CYCLE | -1.82 | -1.59 |  |
|  |  | KREBPATHWAY |  | -1.66 |  |
|  |  | ETCPATHWAY | -1.43 | -1.2 |  |
|  |  | UBIQUINONE_BIOSYNTHESIS | -1.8 | -1.26 |  |
|  |  | GALACTOSE_METABOLISM |  | -1.44 |  |
|  |  | PENTOSE_PHOSPHATE_PATHWAY |  | -1.77 |  |
|  |  | HSA00500_STARCH_AND_SUCROSE_METABOLISM |  | -1.91 |  |
|  |  | HSA00190_OXIDATIVE_PHOSPHORYLATION |  | -1.64 | 1.67 |
|  |  | HSA00790_FOLATE_BIOSYNTHESIS |  |  | 1.89 |
|  | Avoiding immune destruction | HSA04650_NATURAL_KILLER_CELL_MEDIATED_CYTOTOXICITY | 1.67 |  |  |
|  |  | ST_T_CELL_SIGNAL_TRANSDUCTION | 1.24 | -1.47 |  |
|  |  | SIG_BCR_SIGNALING_PATHWAY | 1.56 | -1.61 |  |
|  |  | FCER1PATHWAY | 1.54 |  |  |
|  |  | SIG_PIP3_SIGNALING_IN_B_LYMPHOCYTES |  | -1.75 |  |
|  |  | HSA04612_ANTIGEN_PROCESSING_AND_PRESENTATION |  | -1.85 |  |
| Disrupted liver metabolism | Blood factors | HSA04610_COMPLEMENT_AND_COAGULATION_CASCADES | -2.41 |  | -1.89 |
|  |  | INTRINSICPATHWAY | -1.92 |  | -1.39 |
|  |  | EXTRINSICPATHWAY | -1.57 | 1.57 | -1.55 |
|  |  | FIBRINOLYSISPATHWAY | -1.71 | 1.31 |  |
|  |  | COMPLEMENT_ACTIVATION_CLASSICAL |  | -1.39 |  |
|  |  | CLASSICPATHWAY |  | -1.41 |  |
|  | Hormone regulation | HSA00100_BIOSYNTHESIS_OF_STEROIDS | 1.4 | 2.57 |  |
|  |  | CHOLESTEROL_BIOSYNTHESIS |  | 2.39 |  |
|  |  | BREAST_CANCER_ESTROGEN_SIGNALING |  | 1.67 |  |
|  |  | HSA00150_ANDROGEN_AND_ESTROGEN_METABOLISM | -1.29 | -1.17 |  |
|  | Amino acid metabolism | HSA00910_NITROGEN_METABOLISM |  | -1.91 |  |
|  |  | HSA00530_AMINOSUGARS_METABOLISM | 1.29 | -1.49 |  |
|  |  | HSA00220_UREA_CYCLE_AND_METABOLISM_OF_AMINO_GROUPS |  | -1.5 |  |
|  |  | HSA00340_HISTIDINE_METABOLISM | -1.34 | -1.76 |  |
|  |  | HSA00480_GLUTATHIONE_METABOLISM | -1.36 | -1.33 | -1.47 |
|  |  | HSA00632_BENZOATE_DEGRADATION_VIA_COA_LIGATION | -1.43 | -1.51 | -1.37 |
|  |  | HSA00903_LIMONENE_AND_PINENE_DEGRADATION | -1.44 | -2.11 |  |
|  |  | HSA00360_PHENYLALANINE_METABOLISM | -1.45 | -1.53 |  |
|  |  | HSA00591_LINOLEIC_ACID_METABOLISM | -1.61 |  | -1.39 |
|  |  | HSA00350_TYROSINE_METABOLISM | -1.61 | -1.76 |  |
|  |  | HSA00630_GLYOXYLATE_AND_DICARBOXYLATE_METABOLISM | -1.54 | -1.09 |  |
|  |  | HSA00410_BETA_ALANINE_METABOLISM | -2.63 | -1.81 |  |
|  |  | HSA00252_ALANINE_AND_ASPARTATE_METABOLISM | -1.33 | -1.53 |  |
|  |  | BUTANOATE_METABOLISM | -1.57 | -1.84 |  |
|  |  | HSA00053_ASCORBATE_AND_ALDARATE_METABOLISM | -1.61 | -1.84 |  |
|  |  | LYSINE_DEGRADATION | -1.62 | -1.37 | -1.36 |
|  |  | HSA00680_METHANE_METABOLISM | -1.62 | -1.52 |  |
|  |  | GLYCINE_SERINE_AND_THREONINE_METABOLISM | -1.7 | -1.49 |  |
|  |  | HSA00280_VALINE_LEUCINE_AND_ISOLEUCINE_DEGRADATION | -2.67 | -1.7 |  |
|  |  | TRYPTOPHAN_METABOLISM | -2.17 | -1.95 | -1.36 |
|  |  | HSA00620_PYRUVATE_METABOLISM | -2.36 | -1.99 |  |
|  |  | HSA00640_PROPANOATE_METABOLISM | -1.98 | -2.07 |  |
|  |  | METHIONINE_METABOLISM | -1.12 | -1.41 |  |
|  |  | HSA00450_SELENOAMINO_ACID_METABOLISM |  | -1.59 |  |
|  | Detoxification and xenobiotic metabolism | HSA00641_3_CHLOROACRYLIC_ACID_DEGRADATION | -1.8 | -1.84 |  |
|  |  | HSA00980_METABOLISM_OF_XENOBIOTICS_BY_CYTOCHROME_P450 | -2.48 | -1.59 |  |
|  | Fatty acid metabolism | HSA03320_PPAR_SIGNALING_PATHWAY | -2.71 | -1.56 | -1.54 |
|  |  | HSA00071_FATTY_ACID_METABOLISM | -3.41 | -1.71 | -1.34 |
|  |  | HSA01040_POLYUNSATURATED_FATTY_ACID_BIOSYNTHESIS | -2.87 | -1.5 |  |
|  |  | BETAOXIDATIONPATHWAY | -2.1 | -1.09 |  |
|  |  | FATTY_ACID_BIOSYNTHESIS_PATH_2 | -2 |  |  |
|  |  | HSA00592_ALPHA_LINOLENIC_ACID_METABOLISM | -1.78 |  | -1.34 |
|  |  | HSA00590_ARACHIDONIC_ACID_METABOLISM | -2.15 | -1.11 | -1.47 |
|  |  | HSA00770_PANTOTHENATE_AND_COA_BIOSYNTHESIS | -2.22 |  |  |
|  |  | HSA00561_GLYCEROLIPID_METABOLISM |  | -1.52 |  |
|  | Bile synthesis | HSA00120_BILE_ACID_BIOSYNTHESIS | -2 | -1.53 |  |
