# Supplementary material for: Xmrk, Kras and Myc Transgenic Zebrafish Liver Cancer Models Share Molecular Signatures with Subsets of Human Hepatocellular Carcinoma
Source: PLoS One. 2014 Mar 14;9(3):e91179. doi: 10.1371/journal.pone.0091179 (PMC3954698; doi:10.1371/journal.pone.0091179)
Supplement: Table S6 — Details of pathways differentially expressed in the subgroups of human HCCs which showed significantly correlation with the three zebrafish signatures. (DOCX) [file pone.0091179.s009.docx]

Table S6. Details of pathways differentially expressed in the subgroups of human HCCs which showed significantly correlation with the three zebrafish signatures

| Cluster | NAME | 364_X | 1898_X | 10141_X | 9843_X | 19977_X | 10186_X | 20017_X | 25097_X | 5975_X | 14520_X | 364_K | 1898_K | 10141_K | 9843_K | 19977_K | 10186_K | 20017_K | 25097_K | 5975_K | 14520_K | 364_M | 1898_M | 10141_M | 9843_M | 19977_M | 10186_M | 20017_M | 25097_M | 5975_M | 14520_M |
| --- | --- | --- | --- | --- | --- | --- | --- | --- | --- | --- | --- | --- | --- | --- | --- | --- | --- | --- | --- | --- | --- | --- | --- | --- | --- | --- | --- | --- | --- | --- | --- |
| A | HSA03050_PROTEASOME | 0.967 | 2.92 | 1.65 | 5 | 5 | 2 | 5 | 5 | 2.11 | 5 | 2.35 | 5 | 2.46 | 3.98 | 3.05 | 2.57 | 3.61 | 5 | 3.06 | 5 | 1E-06 | 5 | -0.199 | 4 | 3.3 | 0.325 | 2.49 | 5 | 2.79 | 2.6 |
|  | PROTEASOMEPATHWAY | NaN | 2.62 | 1.72 | 4.05 | 2.53 | 2.78 | 1.78 | 5 | 2.69 | 3.14 | NaN | 5 | 2.23 | 3.1 | 2.86 | 2.53 | 2.08 | 5 | 3.55 | 5 | NaN | 5 | -0.375 | 3.12 | 2.84 | 0.026 | 1.7 | 5 | 3.54 | 1.39 |
|  | HSA00970_AMINOACYL_TRNA_BIOSYNTHESIS | 1.1 | 1.63 | 1.73 | 4.16 | 2.47 | 2.64 | 1.63 | 5 | 2.35 | 4.01 | 1.71 | 3.18 | 2.2 | 2.44 | 5 | 2.05 | 3.46 | 5 | 2.3 | 2.06 | 1E-06 | 1.35 | -0.67 | 2.4 | 3.2 | -0.353 | 5 | 5 | 2.24 | 0.365 |
|  | HSA00190_OXIDATIVE_PHOSPHORYLATION | 0.708 | 2.52 | 0.53 | 5 | 0.723 | 1.7 | 0.722 | 0.658 | 1.57 | 0.63 | 5 | 5 | 2.47 | 5 | 3 | 5 | 5 | 5 | 5 | 5 | 1E-06 | 5 | 0.058 | 5 | 3.39 | 0.274 | 3.45 | 5 | 5 | 5 |
|  | OXIDATIVE_PHOSPHORYLATION | 0.111 | 1.8 | 0.421 | 4.14 | 0.726 | 0.864 | 0.726 | 0.654 | 1.15 | 0.633 | 2.61 | 5 | 2.14 | 3.05 | 2.27 | 2.47 | 3.27 | 5 | 5 | 3 | 1E-06 | 5 | 0.063 | 3.6 | 2.9 | 0.218 | 3.3 | 5 | 5 | 2.58 |
|  | HSA03010_RIBOSOME | 0.967 | 1.08 | 0.632 | 5 | 1.43 | 0.77 | 0.606 | 3.12 | 0.725 | 5 | 1.83 | 3.24 | 1E-06 | 5 | 1.14 | 1E-06 | 0.525 | 1.69 | 1.17 | 1E-06 | 5 | 2.64 | -0.485 | 5 | 5 | -0.232 | 5 | 5 | 1.16 | 5 |
|  | RIBOSOMAL_PROTEINS | 0.848 | 1.85 | 0.381 | 5 | 1.39 | 0.663 | 0.55 | 2.24 | 0.438 | 2.82 | 1.54 | 3.31 | 0.083 | 5 | 1.43 | 0.207 | 0.657 | 1.15 | 0.546 | -0.034 | 5 | 3.26 | -0.491 | 5 | 5 | -0.205 | 5 | 5 | 0.553 | 5 |
|  | TRANSLATION_FACTORS | 0.903 | 2.14 | 0.606 | 2.62 | 1.56 | 0.512 | 1.28 | 0.078 | 1.96 | 2.12 | 2.54 | 2.88 | 0.255 | 1.63 | 1.12 | 0.216 | 1 | 0.356 | 2.32 | 1.93 | 0.086 | 5 | -0.165 | 1.77 | 1.58 | 0.355 | 1.98 | 2 | 2.53 | 2.68 |
|  | HSA04120_UBIQUITIN_MEDIATED_PROTEOLYSIS | 0.693 | 2.49 | NaN | 3.5 | 1.37 | 0.923 | 1.54 | 1.47 | 0.357 | 3.46 | 1.94 | 5 | NaN | 1.87 | 1.03 | 1.46 | 1.08 | 0.37 | 0.422 | 0.59 | 1E-06 | 3.15 | NaN | 1.8 | 1.26 | 0.045 | 1.61 | -0.162 | 0.415 | 0.835 |
|  | RNA_TRANSCRIPTION_REACTOME | 1.78 | 1.39 | 0.227 | 5 | 1.62 | 0.313 | 1.28 | 2.94 | 0.53 | 5 | 1.68 | 1.27 | 0.857 | 2.26 | 2.78 | 0.441 | 3.11 | 2.14 | 0.315 | 1.02 | 1E-06 | 1.45 | -0.159 | 2.31 | 3.52 | 0.163 | 2.46 | 2.42 | 0.302 | 0.921 |
|  | PYRIMIDINE_METABOLISM | 0.879 | 1.14 | 0.408 | 2.86 | 0.905 | 0.748 | 0.875 | 2.25 | 0.767 | 5 | 1.33 | 2.09 | 0.073 | 2.21 | 1.24 | 0.193 | 0.969 | 0.943 | 0.474 | 1.45 | 0.395 | 2.59 | -0.521 | 2.2 | 1.86 | -0.188 | 1.59 | 1.01 | 0.474 | 0.583 |
|  | HSA00240_PYRIMIDINE_METABOLISM | 0.287 | 0.751 | 0.623 | 2.98 | 0.981 | 0.804 | 1.31 | 2.04 | 0.543 | 5 | 0.53 | 1.27 | 0.042 | 3.08 | 1.39 | 0.158 | 1.26 | 1.93 | 0.601 | 2.11 | 1E-06 | 1.2 | -0.754 | 2.91 | 1.37 | -0.092 | 1.19 | 1.69 | 0.615 | 0.389 |
|  | MRNA_PROCESSING_REACTOME | 1.24 | 1.55 | 1.79 | 5 | 3.21 | 1.32 | 2.2 | 5 | 2.3 | 5 | 1.6 | 2.82 | 0.134 | 2.03 | 1.27 | 0.121 | 1.05 | 0.929 | 1.67 | 1E-06 | 1.95 | 2.56 | -0.66 | 2.03 | 1.2 | 0.03 | 1.34 | 0.439 | 1.66 | 0.348 |
|  | UBIQUITIN_MEDIATED_PROTEOLYSIS | NaN | 0.844 | 1.11 | 5 | 0.011 | 0.868 | -0.003 | 3.16 | 0.574 | 3.43 | NaN | 1.72 | 0.109 | 1.82 | 0.123 | 0.401 | -0.001 | 2.05 | 0.547 | 0.802 | NaN | 1.44 | -0.488 | 1.98 | 0.214 | -0.013 | 0.058 | 1.42 | 0.522 | 1.76 |
|  | HSA04110_CELL_CYCLE | 0.083 | 0.846 | 1.64 | 5 | 0.721 | 2.89 | 0.537 | 5 | 1.55 | 5 | 0.173 | 1.25 | 1E-06 | 1.13 | 0.179 | 1E-06 | 0.127 | 0.801 | 0.863 | 0.029 | 1E-06 | 1.03 | -0.489 | 1.12 | 0.431 | 0.044 | 0.326 | -0.021 | 0.805 | 0.56 |
|  | DNA_REPLICATION_REACTOME | 0.055 | 0.48 | 1.11 | 5 | 0.751 | 1.18 | 0.603 | 3.78 | 1.14 | 5 | 0.427 | 0.885 | 1E-06 | 2.03 | 0.16 | 1E-06 | 1E-06 | 0.864 | 0.569 | 0.217 | 1E-06 | 0.936 | -0.263 | 2.06 | 0.436 | 0.111 | 0.114 | 0.072 | 0.582 | 0.679 |
|  | CELL_CYCLE_KEGG | 0.009 | 0.323 | 1.76 | 5 | 0.131 | 2.76 | 0.044 | 5 | 1.08 | 5 | 0.003 | 0.296 | 1E-06 | 0.597 | -0.013 | 1E-06 | -0.042 | 1.15 | 0.319 | 1E-06 | 1E-06 | 0.342 | -0.5 | 0.651 | 0.001 | -0.228 | -0.06 | 0.03 | 0.288 | 0.254 |
|  | G1_TO_S_CELL_CYCLE_REACTOME | 0.004 | 0.289 | 0.588 | 4.08 | 0.257 | 0.545 | 0.31 | 5 | 0.985 | 5 | 0.095 | 0.184 | -0.029 | 1.26 | 0.023 | -0.127 | -0.001 | 0.573 | 0.273 | 1E-06 | 1E-06 | 0.449 | -0.252 | 1.38 | 0.163 | -0.23 | 0.004 | 0.065 | 0.265 | 0.524 |
| B | HSA00020_CITRATE_CYCLE | NaN | 1.51 | 0.028 | 0.202 | 0.692 | 0.082 | 0.621 | -0.947 | 2.01 | 0.07 | NaN | 2.82 | 0.886 | 5 | 1.58 | 0.947 | 3.14 | 2.43 | 2.93 | 3.35 | NaN | 2.6 | 0.07 | 5 | 1.04 | 0.233 | 1.52 | 2.29 | 3.09 | 5 |
|  | KREBS_TCA_CYCLE | 0.474 | 0.671 | 0.167 | 1.28 | 1.12 | 0.157 | 0.912 | -0.435 | 5 | 0.285 | 3.28 | 1.29 | 0.354 | 3.95 | 2.86 | 0.458 | 3.66 | 1.59 | 3.65 | 3.18 | 1E-06 | 1.17 | -0.111 | 5 | 1.75 | 0.272 | 1.35 | 1.27 | 2.99 | 5 |
|  | PROPANOATE_METABOLISM | 0.836 | 0.399 | -0.025 | -0.418 | 0.004 | -0.001 | 0.151 | -2.34 | 0.579 | -0.057 | 2.24 | 1.1 | 0.685 | 5 | 1.98 | 0.398 | 3.23 | 1.01 | 3.18 | 5 | 1E-06 | 1.21 | 0.197 | 5 | 0.57 | 0.307 | 0.495 | 3.92 | 3.01 | 5 |
|  | HSA00640_PROPANOATE_METABOLISM | 0.623 | 0.169 | -0.068 | -0.682 | 0.007 | -0.042 | 0.11 | -2.4 | 0.325 | -0.169 | 1.7 | 0.646 | 0.685 | 5 | 1.43 | 0.612 | 2.37 | 0.397 | 2.59 | 5 | 1E-06 | 0.78 | 0.084 | 5 | 0.411 | 0.312 | 0.368 | 1.71 | 2.71 | 5 |
|  | PYRUVATE_METABOLISM | 1.27 | 0.275 | 0.041 | 0.605 | 0.477 | 1E-06 | 0.558 | -0.706 | 1.33 | 0.101 | 2.61 | 0.65 | 0.088 | 4.01 | 1.46 | 0.051 | 1.68 | 1.43 | 3.73 | 5 | 1E-06 | 0.459 | 0.051 | 3.97 | 0.854 | 0.022 | 0.825 | 1.99 | 3.12 | 3.79 |
|  | HSA00620_PYRUVATE_METABOLISM | 0.857 | 0.123 | 0.031 | 0.267 | 0.326 | -0.04 | 0.44 | -1.03 | 0.649 | 0.103 | 2.64 | 0.281 | 0.081 | 3.36 | 1.21 | 0.021 | 1.53 | 1.03 | 2.72 | 3.72 | 1E-06 | 0.341 | -0.103 | 3.1 | 0.682 | 1E-06 | 0.744 | 1.09 | 2.73 | 4.04 |
|  | HSA00650_BUTANOATE_METABOLISM | 0.106 | 0.045 | -0.492 | -0.282 | 1E-06 | -0.635 | 1E-06 | -2.14 | 0.316 | -0.209 | 1.38 | 0.101 | 0.9 | 3.34 | 1.44 | 0.448 | 1.26 | 1.11 | 1.95 | 4.13 | 1E-06 | 0.019 | 0.16 | 3.44 | 0.437 | 0.267 | 0.094 | 1.33 | 2.25 | 4.3 |
|  | HSA00260_GLYCINE_SERINE_AND_THREONINE_METABOLISM | 0.438 | 0.739 | -0.352 | -0.419 | 0.728 | -0.625 | 0.756 | -1.82 | 0.44 | -0.144 | 1.42 | 1.18 | 1E-06 | 2.58 | 2.13 | -0.038 | 3.54 | 1.5 | 2.16 | 5 | 1E-06 | 1.07 | 0.179 | 2.61 | 1.64 | 0.297 | 1.62 | 1.27 | 2.19 | 5 |
|  | GLYCINE_SERINE_AND_THREONINE_METABOLISM | 0.098 | 0.477 | -0.375 | -0.708 | 0.74 | -0.6 | 0.838 | -1.75 | 0.758 | -0.111 | 0.58 | 0.963 | 0.08 | 1.65 | 1.94 | -0.015 | 3.72 | 0.368 | 2.24 | 4.07 | 1E-06 | 1.16 | 0.196 | 1.71 | 1.66 | 0.15 | 1.52 | 0.372 | 2.45 | 5 |
|  | HSA00220_UREA_CYCLE_AND_METABOLISM_OF_AMINO_GROUPS | 0.652 | 0.476 | -0.431 | 0.577 | 0.72 | -0.011 | 0.754 | -1.07 | 0.421 | 0.745 | 1.66 | 0.88 | 1E-06 | 2.58 | 1.54 | -0.045 | 1.88 | 0.048 | 2.58 | 1.9 | 1E-06 | 0.966 | 0.059 | 2.45 | 1.02 | 0.104 | 1.09 | 0.779 | 2.46 | 2.97 |
|  | HSA00310_LYSINE_DEGRADATION | 0.38 | 0.604 | 0.127 | -1.03 | 0.298 | 1E-06 | 0.298 | -1.11 | 0.558 | -0.215 | 1.65 | 0.895 | 0.53 | 2.91 | 1.78 | 0.391 | 1.38 | 0.192 | 1.93 | 2.86 | 1E-06 | 0.661 | 0.077 | 2.87 | 1.03 | 0.222 | 0.918 | 0.893 | 1.98 | 4.06 |
|  | LYSINE_DEGRADATION | NaN | 0.075 | 0.026 | -0.625 | 0.224 | 1E-06 | 0.256 | -1.45 | 0.57 | -0.171 | NaN | 0.269 | 0.143 | 2.85 | 1.15 | 0.221 | 1.14 | -0.015 | 1.74 | 3.11 | NaN | 0.169 | 0.483 | 2.66 | 0.58 | 0.271 | 0.509 | 0.857 | 2.18 | 3.27 |
|  | HSA00280_VALINE_LEUCINE_AND_ISOLEUCINE_DEGRADATION | 0.013 | 0.481 | -0.672 | -1.56 | 0.022 | -0.664 | 0.281 | -5 | 0.374 | -1.51 | 2.22 | 0.983 | 1.59 | 5 | 2.21 | 0.686 | 3.24 | 1E-06 | 2.96 | 3.5 | 1E-06 | 1.48 | 0.192 | 5 | 1.49 | 0.305 | 1.18 | 5 | 3 | 5 |
|  | VALINE_LEUCINE_AND_ISOLEUCINE_DEGRADATION | 0.08 | 0.4 | -0.307 | -1.02 | 1E-06 | -0.173 | 0.037 | -2.89 | 0.338 | -1.52 | 2.18 | 0.874 | 0.551 | 5 | 1.74 | 0.321 | 3.36 | 0.045 | 2.97 | 3.85 | 1E-06 | 1.04 | 0.184 | 5 | 0.715 | 0.332 | 0.371 | 5 | 3.04 | 5 |
|  | HSA00071_FATTY_ACID_METABOLISM | 0.172 | 0.622 | -0.416 | -2.13 | 0.005 | -1.54 | 1E-06 | -5 | 0.025 | -2.18 | 2.5 | 1.6 | 0.841 | 5 | 1.46 | 0.139 | 3.31 | 0.005 | 1.81 | 5 | 1E-06 | 1.43 | 0.056 | 5 | 1.36 | 0.27 | 0.929 | 5 | 1.78 | 5 |
|  | HSA00380_TRYPTOPHAN_METABOLISM | 0.205 | 1E-06 | -0.418 | 0.098 | 0.451 | -0.359 | 0.595 | -1.45 | 0.146 | -0.173 | 1.35 | -0.019 | 1E-06 | 5 | 1.21 | 1E-06 | 2.41 | 0.394 | 1.04 | 2.34 | 1E-06 | 0.012 | 0.172 | 5 | 1.32 | 0.083 | 0.922 | 3.49 | 1.03 | 5 |
|  | HSA00120_BILE_ACID_BIOSYNTHESIS | 0.102 | 0.3 | -0.654 | -2.26 | 0.146 | -0.947 | 0.271 | -2.9 | 0.069 | -0.654 | 1.94 | 0.76 | 0.186 | 3.73 | 1.66 | 1E-06 | 1.94 | 0.354 | 0.571 | 3.57 | 1E-06 | 0.579 | -0.165 | 5 | 1.12 | 0.057 | 1.07 | 5 | 0.633 | 5 |
|  | TRYPTOPHAN_METABOLISM | 0.178 | 0.017 | -2.85 | -1.59 | -0.004 | -1.51 | -0.007 | -3.81 | 0.078 | -2.19 | 2.63 | 0.129 | 1E-06 | 5 | 0.524 | -0.004 | 1.03 | 0.037 | 1.74 | 3.74 | 1E-06 | 0.053 | 0.446 | 5 | 0.446 | 0.249 | 0.055 | 2.48 | 1.88 | 5 |
|  | HSA00510_N_GLYCAN_BIOSYNTHESIS | 0.857 | 0.782 | NaN | 2.93 | 0.495 | 1.88 | 0.283 | 0.654 | 2.3 | 0.982 | 0.582 | 0.953 | NaN | 0.977 | 0.897 | 2.65 | 0.977 | 3.75 | 5 | 3.76 | 1E-06 | 0.968 | NaN | 0.957 | 1.19 | 0.289 | 0.922 | 1.15 | 5 | 2.28 |
|  | HSA00100_BIOSYNTHESIS_OF_STEROIDS | 0.058 | 0.422 | 0.008 | 0.977 | 0.035 | 0.324 | 0.139 | -0.077 | 0.372 | 0.185 | 2.51 | 1.12 | 2.27 | 0.219 | 0.908 | 2.27 | 0.805 | 3.37 | 1.43 | 5 | 1E-06 | 0.414 | 0.246 | 0.232 | 0.986 | 2.23 | 0.872 | 1.01 | 1.35 | 3.91 |
|  | HSA04610_COMPLEMENT_AND_COAGULATION_CASCADES | -0.007 | 0.146 | -3 | -0.184 | 1E-06 | 2 | 1E-06 | -5 | 0.474 | -3.6 | 1.95 | 0.295 | 0.109 | 3.38 | 0.843 | 1E-06 | 2.08 | 0.03 | 2.93 | 5 | 1E-06 | 0.23 | 0.489 | 2.76 | 0.549 | 0.231 | 0.974 | 0.113 | 3.03 | 2.74 |
|  | BLOOD_CLOTTING_CASCADE | NaN | 0.107 | -2.11 | -0.542 | -0.018 | -2.09 | -0.011 | -2.4 | 0.168 | -1.27 | NaN | 0.158 | 0.198 | 2.16 | 0.379 | 1E-06 | 0.599 | 1E-06 | 1.27 | 2.48 | NaN | 0.002 | 0.623 | 2.27 | 0.212 | 0.302 | 0.067 | 0.015 | 1.34 | 2.28 |
|  | INTRINSICPATHWAY | 0.013 | -0.071 | -2.09 | -1.34 | -0.096 | 2 | -0.031 | -5 | 0.444 | -2.2 | 1.31 | -0.321 | 0.303 | 0.475 | 0.123 | 0.125 | 0.311 | 1E-06 | 1.32 | 3.1 | 1E-06 | 0.033 | 0.58 | 0.474 | 0.006 | 0.278 | 1E-06 | -0.097 | 1.24 | 1.29 |
|  | HSA00350_TYROSINE_METABOLISM | 0.049 | -0.086 | -1.48 | 0.253 | 0.355 | -0.951 | 0.271 | -0.818 | 0.174 | -0.316 | 0.21 | -0.05 | -0.189 | 1.56 | 0.909 | -0.192 | 1.65 | 0.437 | 0.424 | 1 | 1E-06 | 1E-06 | -0.203 | 1.62 | 1.66 | -0.014 | 1.29 | 1.36 | 0.454 | 2.95 |
|  | HSA00340_HISTIDINE_METABOLISM | 0.12 | -0.016 | -0.301 | 0.272 | 0.101 | -0.04 | 0.262 | -0.842 | 0.019 | 0.058 | 0.793 | -0.015 | 1E-06 | 1.51 | 0.473 | -0.007 | 0.968 | 0.496 | 0.261 | 1.17 | 1E-06 | 0.016 | -0.061 | 1.45 | 0.708 | -0.124 | 0.654 | 0.986 | 0.265 | 2.58 |
|  | HISTIDINE_METABOLISM | 0.427 | 0.134 | -1.48 | -0.747 | 1E-06 | -0.64 | 1E-06 | -0.815 | 0.182 | -0.177 | 1.47 | 0.277 | 1E-06 | 1.11 | 0.458 | -0.105 | 0.793 | 0.359 | 1.17 | 0.909 | 1E-06 | 0.297 | 0.076 | 1.09 | 0.423 | 0.038 | 0.095 | 0.402 | 1.12 | 2.27 |
|  | PHENYLALANINE_METABOLISM | 0.046 | 1E-06 | -2.15 | -0.241 | -0.002 | -1.14 | -0.011 | -1.01 | 0.078 | -1.19 | 0.889 | -0.003 | -0.349 | 1.11 | 0.371 | -0.335 | 0.756 | 0.779 | 0.314 | -0.057 | 1E-06 | -0.002 | -0.208 | 1.05 | 0.525 | -0.003 | 0.092 | 1.01 | 0.302 | 2.44 |
|  | HSA00360_PHENYLALANINE_METABOLISM | 0.049 | 1E-06 | -2.13 | -0.428 | 0.098 | -1.1 | 0.029 | -0.68 | 0.068 | -0.599 | 0.212 | 1E-06 | -0.214 | 0.409 | 0.524 | -0.356 | 0.664 | 1E-06 | 0.217 | 0.123 | 1E-06 | 1E-06 | -0.157 | 0.384 | 0.524 | -0.06 | 0.463 | 0.07 | 0.206 | 2.39 |
|  | TYROSINE_METABOLISM | 0.087 | 1E-06 | -3.16 | 2 | -0.21 | -3.08 | -0.028 | -5 | 0.005 | -3.07 | 0.726 | 1E-06 | -0.326 | 1.98 | 0.208 | -0.516 | 0.685 | 1E-06 | 0.187 | 0.571 | 1E-06 | 1E-06 | -0.163 | 1.76 | 0.333 | 0.011 | 0.094 | 0.775 | 0.187 | 2.91 |
| C | HSA00252_ALANINE_AND_ASPARTATE_METABOLISM | 0.65 | 0.124 | 0.072 | 0.143 | 1E-06 | 0.163 | 0.199 | -0.754 | 1.56 | 0.839 | 1.7 | 0.225 | 0.575 | 2.27 | 0.603 | 0.684 | 1.21 | 0.742 | 0.913 | 2.89 | 1E-06 | 0.338 | -0.094 | 2.35 | 0.404 | 0.227 | 0.503 | 1.98 | 0.886 | 5 |
|  | HSA00330_ARGININE_AND_PROLINE_METABOLISM | -0.002 | 0.25 | -0.21 | 0.184 | 0.101 | -0.06 | 0.18 | -0.553 | 0.416 | -0.026 | 0.573 | 0.224 | 0.042 | 1.94 | 0.879 | 1E-06 | 0.661 | 1.11 | 1.23 | 1.7 | 1E-06 | 0.345 | 0.179 | 2.15 | 0.527 | 0.256 | 0.383 | 2.71 | 1.21 | 5 |
|  | ARGININE_AND_PROLINE_METABOLISM | 0.476 | 0.148 | -0.521 | 0.382 | -0.003 | -0.114 | -0.012 | -1.02 | 0.431 | 0.061 | 1.61 | 0.345 | 1E-06 | 2.14 | 0.373 | -0.002 | 0.454 | 0.356 | 2.48 | 2.09 | 1E-06 | 0.448 | 0.259 | 2.09 | 0.084 | 0.164 | 0.031 | 1.53 | 2.76 | 4.02 |
|  | HSA00150_ANDROGEN_AND_ESTROGEN_METABOLISM | -0.078 | -0.14 | -0.272 | 0.145 | 1E-06 | 1E-06 | 1E-06 | -0.551 | 0.028 | -0.062 | 0.334 | -0.161 | 0.08 | 2.41 | 0.491 | 1E-06 | 0.566 | 0.738 | 0.081 | 2.47 | -0.075 | 1E-06 | 0.071 | 2.31 | 0.415 | 0.159 | 0.118 | 1.99 | 0.085 | 2.27 |
|  | PORPHYRIN_AND_CHLOROPHYLL_METABOLISM | NaN | 0.392 | 0.383 | 0.664 | 0.375 | 0.278 | 0.543 | 0.479 | 0.504 | 0.817 | NaN | 0.877 | 0.36 | 1.54 | 0.888 | 0.349 | 1.33 | 1.59 | 1.17 | 3.09 | NaN | 0.662 | -0.492 | 1.72 | 0.131 | -0.484 | 0.067 | 0.156 | 1.16 | 0.7 |
|  | HSA00860_PORPHYRIN_AND_CHLOROPHYLL_METABOLISM | 0.02 | 0.401 | 0.076 | 0.575 | 1E-06 | 0.1 | 0.137 | -0.339 | 0.391 | 0.255 | 1.19 | 0.864 | 0.347 | 2.85 | 0.85 | 0.201 | 0.701 | 1.12 | 0.98 | 4.1 | 1E-06 | 0.67 | -0.177 | 2.67 | -0.005 | -0.308 | 1E-06 | 0.528 | 0.984 | 1.38 |
|  | GLUTATHIONE_METABOLISM | 0.053 | 0.415 | -0.344 | 0.397 | 0.166 | 0.031 | 1E-06 | 0.432 | 0.429 | 0.541 | 0.441 | 0.914 | 0.189 | 1.76 | 0.808 | 0.02 | 0.628 | 3.42 | 0.856 | 3.45 | 1E-06 | 0.346 | 0.037 | 1.8 | 0.251 | 0.154 | 0.26 | 0.81 | 0.807 | 2.13 |
|  | HSA00480_GLUTATHIONE_METABOLISM | 0.017 | 0.396 | -0.493 | 0.06 | 1E-06 | 1E-06 | -0.013 | 0.251 | 0.546 | 0.28 | 0.433 | 0.918 | 0.189 | 1.08 | 0.373 | 0.018 | 0.388 | 3.45 | 1.33 | 5 | -0.081 | 0.258 | 0.068 | 1.04 | 0.213 | 0.237 | 0.089 | 0.801 | 1.29 | 2.37 |
|  | HSA00251_GLUTAMATE_METABOLISM | 0.053 | 0.106 | 0.002 | 0.174 | 0.227 | 0.103 | 1E-06 | -0.724 | 0.775 | 0.41 | 0.395 | 0.185 | 0.666 | 0.613 | 0.936 | 0.681 | 0.629 | 1.1 | 1.03 | 3.04 | 1E-06 | 0.081 | -0.091 | 0.559 | 1.01 | 0.267 | 0.637 | 0.628 | 1.08 | 3.49 |
|  | GLUTAMATE_METABOLISM | 0.053 | 1E-06 | 0.029 | 0.082 | 0.449 | 0.141 | 0.138 | -0.672 | 0.617 | 0.455 | 0.431 | 1E-06 | 0.72 | 0.824 | 1.14 | 0.924 | 1.01 | 1.08 | 0.678 | 2.83 | 1E-06 | 1E-06 | -0.095 | 0.831 | 0.978 | 0.312 | 0.653 | 0.74 | 0.743 | 2.91 |
|  | GLYCOLYSIS_AND_GLUCONEOGENESIS | 0.876 | 0.15 | 0.03 | 2.02 | 0.336 | 0.05 | 0.14 | 0.337 | 0.55 | 0.612 | 3.46 | 0.3 | 0.25 | 0.493 | 0.622 | 0.41 | 1.06 | 1.3 | 1.3 | 2.21 | 1E-06 | 0.289 | 0.044 | 0.473 | 0.217 | 0.068 | 0.467 | 0.403 | 1.3 | 1.38 |
|  | GLUCONEOGENESIS | 0.438 | 0.095 | -0.157 | 0.98 | 0.082 | -0.03 | 1E-06 | 0.336 | 0.195 | 0.398 | 2.56 | 0.363 | 0.058 | 1.14 | 0.499 | 1E-06 | 0.815 | 1.63 | 0.945 | 2.87 | 1E-06 | 0.365 | 0.072 | 1.18 | 0.099 | 0.222 | 0.114 | 0.224 | 0.855 | 0.602 |
|  | GLYCOLYSIS | 0.412 | 0.094 | -0.177 | 1.02 | 0.073 | -0.032 | 1E-06 | 0.338 | 0.184 | 0.393 | 2.55 | 0.343 | 0.059 | 1.12 | 0.52 | 1E-06 | 0.778 | 1.68 | 0.922 | 2.69 | 1E-06 | 0.354 | 0.074 | 1.19 | 0.085 | 0.226 | 0.11 | 0.235 | 0.927 | 0.615 |
|  | HSA00010_GLYCOLYSIS_AND_GLUCONEOGENESIS | 0.45 | 0.094 | -0.074 | 0.754 | 0.176 | -0.031 | 0.062 | 0.188 | 0.069 | 0.201 | 2.42 | 0.272 | 0.041 | 0.903 | 0.515 | 1E-06 | 0.876 | 0.941 | 0.443 | 1.83 | 1E-06 | 0.346 | 0.028 | 0.937 | 0.155 | 0.193 | 0.212 | 0.064 | 0.456 | 0.619 |
|  | HSA00500_STARCH_AND_SUCROSE_METABOLISM | 0.458 | 0.193 | -0.764 | 0.384 | 0.824 | -0.037 | 0.842 | -0.658 | 0.147 | 0.632 | 1.36 | 0.079 | 0.041 | 1.07 | 0.897 | -0.005 | 1.5 | 0.808 | 0.448 | 2.38 | 1E-06 | 0.059 | 0.005 | 1.03 | 0.435 | 0.002 | 0.762 | 0.124 | 0.422 | 0.602 |
|  | STARCH_AND_SUCROSE_METABOLISM | 0.857 | 0.004 | -0.421 | 0.404 | 0.151 | -0.007 | 0.323 | -0.379 | 0.047 | 0.817 | 1.39 | -0.001 | 0.248 | 0.177 | 0.156 | 1E-06 | 0.771 | 1.76 | 0.252 | 3.37 | 1E-06 | 0.057 | 0.078 | 0.203 | 0.025 | 0.002 | 0.121 | -0.089 | 0.266 | 0.674 |
| D | MTORPATHWAY | 0.127 | 1.03 | 0.541 | 0.507 | 1.85 | 0.472 | 1.24 | -0.333 | 0.932 | 0.851 | 0.12 | 0.952 | 0.143 | 1.55 | 1.71 | 0.153 | 2.07 | -0.225 | 0.409 | 2.06 | 1E-06 | 1.44 | -0.45 | 1.57 | 1.24 | -0.19 | 2.53 | 0.44 | 0.382 | 1.77 |
|  | EIF4PATHWAY | 0.246 | 0.478 | 0.387 | 0.162 | 1.24 | 0.285 | 1.03 | -0.682 | 0.571 | 0.947 | 0.388 | 0.44 | 0.148 | 1.56 | 1.44 | 0.18 | 1.63 | -0.089 | 0.497 | 2.46 | 1E-06 | 0.357 | -0.445 | 1.71 | 1.18 | -0.094 | 1.83 | 0.015 | 0.459 | 0.859 |
|  | PPARAPATHWAY | 0.049 | 0.403 | 0.001 | -0.027 | 1.44 | 0.019 | 1.29 | -0.827 | 0.761 | 0.241 | 0.108 | 0.354 | 0.076 | 1.64 | 2.11 | 1E-06 | 2.17 | -0.016 | 1.87 | 1.14 | 1E-06 | 0.339 | 0.059 | 1.71 | 0.972 | 0.219 | 0.979 | -0.182 | 2.15 | 1.24 |
|  | HSA00790_FOLATE_BIOSYNTHESIS | 0.747 | 0.124 | -0.192 | 0.428 | 0.842 | 1E-06 | 0.779 | 0.022 | 0.792 | 0.427 | 0.024 | 0.074 | 1E-06 | 2.04 | 0.908 | 1E-06 | 1.2 | 0.499 | 1.07 | 0.988 | 1E-06 | 0.061 | 1E-06 | 2.03 | 0.584 | 0.032 | 1.22 | 0.651 | 1.09 | 0.693 |
|  | PURINE_METABOLISM | 0.442 | 1.05 | 0.377 | 2.2 | 0.786 | 0.528 | 0.697 | 1.43 | 0.442 | 1.38 | 0.928 | 1.06 | 0.105 | 0.533 | 0.912 | 0.2 | 1.06 | 1.7 | 0.589 | 0.644 | 0.072 | 0.926 | -0.457 | 0.55 | 1.67 | 0.036 | 1.5 | 1.98 | 0.548 | 0.406 |
|  | HSA00230_PURINE_METABOLISM | 0.061 | 0.474 | 0.32 | 2.27 | 0.802 | 0.289 | 0.848 | 1.05 | 0.36 | 1.32 | 0.092 | 0.611 | 1E-06 | 0.964 | 0.905 | 1E-06 | 1.23 | 1.57 | 0.315 | 0.591 | 1E-06 | 0.341 | -0.485 | 0.952 | 0.859 | -0.034 | 0.921 | 1.19 | 0.329 | 0.262 |
|  | ST_PHOSPHOINOSITIDE_3_KINASE_PATHWAY | 0.066 | 0.201 | 0.64 | 1.53 | 2.16 | 0.692 | 2.14 | 0.834 | 0.934 | 1.25 | 0.336 | 0.441 | 0.37 | 0.519 | 0.942 | 0.525 | 1.66 | 0.032 | 0.621 | 0.019 | 1E-06 | 0.243 | -0.161 | 0.528 | 0.417 | 0.103 | 1.3 | -0.799 | 0.633 | -0.011 |
|  | RHOPATHWAY | 0.249 | 0.623 | 0.421 | 0.899 | 1.18 | 0.158 | 1.28 | 1.13 | 1.38 | 0.58 | 1.27 | 0.866 | 1E-06 | -0.303 | 0.949 | 1E-06 | 0.991 | -0.177 | 1.19 | -0.025 | 1E-06 | 0.421 | 0.001 | -0.298 | 1.18 | 1E-06 | 1.96 | -0.775 | 1.27 | -0.279 |
|  | VEGFPATHWAY | 0.115 | 1.48 | 0.844 | 1.4 | 0.856 | 0.39 | 0.798 | 0.191 | 1.16 | 0.834 | 0.059 | 1.26 | 0.259 | 0.204 | 0.609 | 0.34 | 1.5 | 0.035 | 2 | 0.378 | 1E-06 | 2.21 | -0.53 | 0.189 | 0.277 | -0.41 | 0.754 | -0.401 | 2.06 | 0.191 |
|  | HSA04130_SNARE_INTERACTIONS_IN_VESICULAR_TRANSPORT | 0.266 | 0.749 | 0.106 | 2.02 | 0.968 | 0.048 | 0.835 | 1.33 | 1.12 | 0.797 | 0.344 | 1.19 | 0.012 | 1.1 | 0.916 | 1E-06 | 0.907 | 0.84 | 1.45 | 0.857 | 1E-06 | 1.1 | 0.035 | 1.04 | 0.279 | 0.025 | 0.472 | -0.291 | 1.3 | -0.004 |
|  | IGF1MTORPATHWAY | 0.133 | 0.729 | 0.145 | 0.44 | 0.911 | 0.219 | 0.904 | -0.747 | 0.969 | 0.456 | 1E-06 | 1.12 | 0.039 | 0.944 | 0.592 | 0.106 | 1.06 | -0.18 | 0.603 | 0.826 | 1E-06 | 1.17 | -0.526 | 0.944 | 0.332 | -0.333 | 0.504 | 0.36 | 0.548 | 0.924 |
|  | ECMPATHWAY | NaN | 0.468 | 0.134 | -0.058 | 0.721 | -0.003 | 0.52 | -0.105 | 1.14 | 0.488 | NaN | 0.719 | 1E-06 | 0.612 | 0.916 | 1E-06 | 0.964 | -0.21 | 1.44 | 1.28 | NaN | 0.666 | -0.381 | 0.606 | 0.437 | -0.328 | 1.12 | -0.733 | 1.46 | 0.13 |
|  | CXCR4PATHWAY | 0.914 | 0.254 | 1E-06 | 0.069 | 0.489 | 0.001 | 0.495 | 0.518 | 0.936 | 0.745 | 0.293 | 0.183 | -0.003 | 0.399 | 0.479 | 1E-06 | 0.969 | 1E-06 | 1.63 | 0.64 | 1E-06 | 0.38 | -0.561 | 0.37 | 0.304 | -0.951 | 0.651 | -0.441 | 1.52 | 0.109 |
|  | HSA04520_ADHERENS_JUNCTION | 0.014 | 0.411 | 0.074 | 0.458 | 0.738 | 0.079 | 0.388 | 0.31 | 0.716 | 0.503 | 0.215 | 0.857 | 1E-06 | 0.019 | 0.246 | 1E-06 | 0.493 | -0.142 | 1.32 | 1E-06 | 1E-06 | 0.347 | -0.163 | 0.018 | 1.21 | 0.107 | 1.05 | -0.069 | 1.3 | -0.021 |
|  | NTHIPATHWAY | NaN | 0.353 | -0.68 | 0.413 | 0.379 | -0.569 | 0.139 | -0.034 | 1.13 | 0.026 | NaN | 0.405 | -0.004 | 0.067 | 0.159 | -0.113 | 0.096 | -0.332 | 1.57 | 0.003 | NaN | 0.14 | -0.227 | 0.06 | 0.455 | -0.409 | 0.373 | -0.318 | 1.46 | -0.029 |
|  | INTEGRINPATHWAY | 0.175 | 0.422 | 0.083 | 0.9 | 0.717 | 0.114 | 0.515 | 0.407 | 1.08 | 1.03 | 0.664 | 0.507 | 1E-06 | -0.296 | 0.178 | 1E-06 | 0.299 | -0.24 | 1.24 | 0.734 | 1E-06 | 0.214 | -0.45 | -0.305 | 0.408 | -0.326 | 0.748 | -1.61 | 1.24 | -0.764 |
|  | MAPKPATHWAY | 0.083 | 0.613 | 0.032 | 0.909 | 0.881 | 0.154 | 0.615 | 0.818 | 0.219 | 0.831 | 0.107 | 0.641 | 1E-06 | 0.019 | 0.696 | 1E-06 | 0.692 | -0.223 | 0.08 | 1E-06 | 1E-06 | 0.514 | -0.441 | 0.017 | 0.419 | -0.402 | 0.918 | -2.34 | 0.084 | -0.207 |
|  | ST_INTEGRIN_SIGNALING_PATHWAY | 0.089 | 0.272 | 0.129 | 0.317 | 0.968 | 0.268 | 0.899 | 0.231 | 0.413 | 0.668 | 0.358 | 0.183 | 1E-06 | -0.161 | 0.192 | 1E-06 | 0.625 | -0.334 | 0.16 | -0.176 | 1E-06 | 0.175 | -0.188 | -0.142 | 0.48 | -0.357 | 0.821 | -2.19 | 0.155 | -0.538 |
|  | PTDINSPATHWAY | 0.249 | 0.145 | 1.08 | 0.607 | 0.941 | 0.971 | 0.75 | 0.981 | 0.447 | 0.384 | -0.028 | 0.24 | 0.325 | 0.145 | 0.585 | 0.606 | 0.903 | -0.038 | 0.186 | 1E-06 | 1E-06 | 0.31 | -0.206 | 0.156 | 0.133 | -0.063 | 0.39 | -0.745 | 0.186 | -0.158 |
|  | ERKPATHWAY | -0.033 | 1E-06 | 0.127 | 0.443 | 0.813 | 0.062 | 0.535 | 1.49 | 0.183 | 0.777 | 0.012 | 0.297 | 0.088 | -0.045 | 0.441 | 0.13 | 0.578 | -0.035 | 0.292 | 0.221 | 1E-06 | 0.291 | 0.051 | -0.054 | 0.281 | 0.234 | 0.913 | -0.788 | 0.292 | -0.086 |
|  | ST_P38_MAPK_PATHWAY | 0.914 | 0.416 | 1E-06 | 1.34 | 0.578 | 1E-06 | 0.173 | 0.913 | 0.413 | 1.01 | 0.396 | 0.246 | -0.154 | 0.082 | 0.439 | -0.123 | 0.457 | 1E-06 | 0.109 | 1E-06 | 1E-06 | 0.351 | 0.058 | 0.094 | 0.33 | -0.09 | 0.47 | -0.169 | 0.114 | -0.08 |
|  | FMLPPATHWAY | -0.07 | 0.125 | -0.177 | 1.28 | 0.149 | -0.063 | 0.067 | 1.07 | 0.048 | 0.66 | -0.157 | 0.171 | -0.158 | 0.165 | 0.073 | -0.02 | 0.072 | 0.243 | 0.16 | -0.177 | 1E-06 | 0.145 | -0.484 | 0.153 | 0.206 | -0.348 | 0.286 | -0.318 | 0.169 | -0.069 |
|  | HSA04012_ERBB_SIGNALING_PATHWAY | -0.02 | 0.124 | 0.059 | 0.284 | 0.805 | 0.156 | 0.638 | 0.533 | 0.09 | 0.605 | -0.148 | 0.068 | 0.012 | 0.028 | 0.277 | 1E-06 | 0.594 | -0.096 | 0.111 | -0.123 | 1E-06 | 0.108 | -0.66 | 0.031 | 0.364 | -0.396 | 0.822 | -0.747 | 0.113 | -0.111 |
|  | SIG_PIP3_SIGNALING_IN_B_LYMPHOCYTES | -0.015 | 0.281 | 0.13 | 0.648 | 0.493 | 0.325 | 0.378 | 0.679 | 0.041 | 0.433 | -0.057 | 0.593 | 0.049 | 0.13 | 0.253 | 0.079 | 0.164 | -0.037 | -0.006 | -0.383 | 1E-06 | 0.356 | -0.502 | 0.126 | 0.268 | -0.429 | 0.367 | -0.78 | -0.006 | -0.074 |
|  | ST_ADRENERGIC | 0.066 | 0.421 | 0.028 | -0.279 | 0.278 | 1E-06 | 0.038 | 0.149 | 0.004 | 0.038 | 0.056 | 0.161 | 0.109 | 0.16 | 0.29 | 0.02 | 0.16 | -0.079 | 0.063 | 0.066 | 1E-06 | 0.105 | -0.297 | 0.157 | 0.27 | -0.27 | 0.485 | -0.735 | 0.066 | -0.016 |
|  | IL2PATHWAY | NaN | 0.243 | 1E-06 | 2.33 | 0.636 | -0.011 | 0.583 | 0.809 | 0.253 | 0.001 | NaN | 0.177 | -0.155 | 0.006 | 0.066 | -0.027 | 0.036 | -0.08 | 0.252 | -0.73 | NaN | 0.74 | -0.329 | 0.007 | 0.215 | -0.174 | 0.427 | -0.759 | 0.251 | -0.625 |
|  | BCRPATHWAY | -0.079 | 0.159 | 1E-06 | 1.97 | 0.709 | 1E-06 | 0.572 | 0.97 | 0.143 | 0.046 | -0.492 | 0.175 | -0.09 | 0.037 | 0.177 | 1E-06 | 0.097 | -0.034 | 0.068 | -0.728 | 1E-06 | 0.343 | -0.502 | 0.042 | 0.236 | -0.389 | 0.26 | -0.783 | 0.076 | -0.18 |
|  | CSKPATHWAY | -0.051 | 1E-06 | 0.029 | 0.607 | 1.16 | 0.109 | 0.778 | 0.315 | 0.194 | 0.002 | -0.058 | -0.022 | -0.135 | 0.031 | 0.519 | -0.026 | 0.392 | -0.092 | 0.208 | -0.812 | 1E-06 | 1E-06 | -0.234 | 0.026 | 0.434 | -0.403 | 0.632 | -0.578 | 0.193 | 1E-06 |
|  | AMIPATHWAY | -0.043 | 1E-06 | 0.03 | 0.606 | 1.23 | 0.108 | 0.757 | 0.287 | 0.171 | 1E-06 | -0.066 | -0.02 | -0.142 | 0.032 | 0.497 | -0.04 | 0.356 | -0.095 | 0.216 | -0.812 | 1E-06 | 1E-06 | -0.237 | 0.027 | 0.426 | -0.416 | 0.637 | -0.541 | 0.222 | 1E-06 |
|  | GPCRPATHWAY | 0.463 | 1E-06 | 0.028 | 0.506 | 0.751 | 0.117 | 0.523 | 0.431 | 0.144 | 0.308 | 0.13 | 1E-06 | 1E-06 | -0.006 | 0.456 | 1E-06 | 0.333 | -0.096 | 0.165 | -0.625 | 1E-06 | 0.386 | -0.489 | -0.007 | 0.286 | -0.243 | 0.366 | -0.279 | 0.171 | -0.004 |
|  | VIPPATHWAY | 0.05 | 0.035 | -0.173 | 0.681 | 0.698 | -0.035 | 0.269 | 0.233 | 0.189 | 0.017 | 0.161 | -0.012 | -0.025 | 0.026 | 0.469 | -0.069 | 0.487 | -0.039 | 0.186 | -0.886 | 1E-06 | 0.106 | -0.089 | 0.025 | 0.148 | -0.273 | 0.141 | -0.234 | 0.189 | 0.021 |
|  | HSA04664_FC_EPSILON_RI_SIGNALING_PATHWAY | 0.085 | -0.369 | -0.104 | 0.444 | 0.634 | -0.057 | 0.52 | 0.779 | 0.048 | 0.108 | -0.029 | -0.547 | 1E-06 | -0.042 | 0.404 | -0.053 | 0.374 | -0.04 | 0.007 | -0.833 | 1E-06 | -0.578 | -0.203 | -0.054 | 0.298 | -0.411 | 0.526 | -0.288 | 0.007 | -0.523 |
|  | ST_FAS_SIGNALING_PATHWAY | 0.117 | 1E-06 | 0.235 | 2.37 | 0.858 | 0.274 | 0.607 | 0.872 | 0.674 | 0.695 | 0.025 | -0.023 | 0.038 | 0.943 | 0.897 | 0.019 | 0.654 | 1E-06 | 0.584 | 0.067 | 1E-06 | 0.08 | -0.049 | 0.942 | 0.98 | -0.082 | 1.28 | -0.703 | 0.615 | -0.079 |
|  | IL2RBPATHWAY | 0.007 | 0.136 | 1E-06 | 0.91 | 0.98 | 1E-06 | 0.775 | 0.84 | 0.57 | 0.741 | 1E-06 | 0.044 | -0.032 | 1.02 | 0.375 | -0.034 | 0.626 | -0.036 | 0.686 | -0.006 | 1E-06 | 0.097 | -0.503 | 1.07 | 0.426 | -0.403 | 0.761 | -0.405 | 0.662 | -0.157 |
|  | HSA05212_PANCREATIC_CANCER | 0.116 | 0.417 | 0.067 | 1.88 | 0.996 | 1E-06 | 0.778 | 1.4 | 0.684 | 0.777 | -0.078 | 0.725 | 1E-06 | 0.159 | 0.5 | 1E-06 | 0.649 | 1E-06 | 0.608 | -0.128 | 1E-06 | 0.246 | -0.509 | 0.173 | 0.571 | -0.403 | 0.934 | -0.284 | 0.635 | -0.288 |
|  | ST_ERK1_ERK2_MAPK_PATHWAY | 0.052 | 0.137 | 0.004 | 1.96 | 0.991 | 0.028 | 0.779 | 1.05 | 0.352 | 1.09 | 0.001 | 0.018 | 1E-06 | 0.018 | 0.479 | 1E-06 | 0.776 | 1E-06 | 0.246 | 0.225 | 1E-06 | 1E-06 | -0.119 | 0.023 | 0.332 | -0.195 | 1.08 | -0.17 | 0.265 | -0.09 |
|  | AT1RPATHWAY | 0.137 | 0.122 | 0.06 | 1.31 | 0.997 | 0.154 | 0.906 | 0.43 | 0.461 | 0.797 | 0.433 | 0.174 | 0.005 | 0.476 | 0.876 | 0.092 | 1.1 | -0.29 | 0.499 | 0.174 | 1E-06 | 0.204 | -0.504 | 0.428 | 0.501 | -0.405 | 0.964 | -0.544 | 0.473 | -0.194 |
|  | HSA04210_APOPTOSIS | 0.064 | 0.211 | 0.03 | 1.61 | 0.718 | 0.048 | 0.559 | 0.312 | 0.575 | 0.018 | 0.036 | 0.041 | 1E-06 | 0.336 | 0.877 | 1E-06 | 0.966 | 0.026 | 0.546 | 0.132 | 1E-06 | 1E-06 | -0.481 | 0.362 | 0.467 | -0.712 | 0.583 | -0.203 | 0.515 | -0.076 |
|  | TOLLPATHWAY | 0.157 | 0.29 | -0.67 | 0.686 | 0.83 | -0.043 | 0.619 | -0.001 | 0.522 | 0.03 | 0.451 | 0.328 | 1E-06 | 0.525 | 0.901 | 1E-06 | 1.51 | 1E-06 | 0.423 | 0.167 | 1E-06 | 0.338 | 0.188 | 0.554 | 0.959 | 0.032 | 1.08 | -0.401 | 0.415 | -0.079 |
|  | METPATHWAY | 0.052 | 0.225 | 0.136 | 0.371 | 0.78 | 0.009 | 0.782 | 0.529 | 0.613 | 0.897 | 0.027 | 0.411 | 0.014 | 0.765 | 0.584 | 0.011 | 1.16 | -0.056 | 0.719 | 0.757 | 1E-06 | 0.484 | -0.513 | 0.699 | 0.529 | -0.407 | 0.916 | -0.747 | 0.691 | -0.003 |
|  | ST_DICTYOSTELIUM_DISCOIDEUM_CAMP_CHEMOTAXIS_PATHWAY | 0.011 | 0.42 | 0.02 | 0.241 | 0.841 | 0.064 | 0.643 | 0.187 | 0.351 | 0.596 | 0.012 | 0.671 | 1E-06 | 0.827 | 0.9 | 1E-06 | 0.999 | 1E-06 | 0.513 | 0.181 | 1E-06 | 0.455 | -0.202 | 0.878 | 0.439 | -0.363 | 0.914 | 0.142 | 0.504 | 0.22 |
|  | PDGFPATHWAY | 0.11 | 0.473 | 1E-06 | 0.457 | 0.93 | 1E-06 | 0.776 | 0.185 | 0.433 | 0.437 | 1E-06 | 0.756 | 1E-06 | 0.415 | 0.588 | -0.005 | 0.794 | -0.167 | 0.493 | -0.004 | 1E-06 | 1.02 | -0.455 | 0.412 | 0.413 | -0.429 | 0.85 | -0.754 | 0.496 | -0.175 |
|  | BIOPEPTIDESPATHWAY | 0.688 | 0.082 | 1E-06 | 0.415 | 0.464 | -0.163 | 0.441 | 0.535 | 0.444 | 0.114 | 0.178 | 0.288 | -0.17 | 0.293 | 0.171 | -0.107 | 0.08 | 0.032 | 0.588 | 1E-06 | 1E-06 | 0.052 | 0.068 | 0.289 | 0.441 | 0.125 | 0.918 | -0.237 | 0.589 | -0.19 |
|  | ST_GA13_PATHWAY | 0.321 | 0.043 | 0.01 | 0.228 | 0.968 | 0.016 | 0.65 | 0.131 | 0.445 | -0.123 | 0.73 | 0.01 | 1E-06 | 0.094 | 0.837 | 1E-06 | 0.69 | -0.034 | 0.421 | -0.114 | 1E-06 | 0.001 | 0.034 | 0.099 | 0.453 | -0.277 | 0.872 | -1.16 | 0.415 | -0.045 |
|  | MCALPAINPATHWAY | 0.355 | 0.143 | 0.32 | 0.008 | 0.388 | 0.155 | 0.257 | 0.064 | 0.626 | 0.072 | 0.452 | 0.176 | 0.135 | 1E-06 | 0.452 | 0.132 | 0.577 | 1E-06 | 0.521 | -0.093 | 1E-06 | 0.213 | 0.069 | 1E-06 | 0.095 | 0.032 | 0.509 | -0.547 | 0.523 | -0.039 |
|  | HSA04150_MTOR_SIGNALING_PATHWAY | 0.05 | 1E-06 | 0.002 | 0.052 | 1.46 | 0.148 | 1.04 | 0.283 | 0.17 | 0.542 | -0.319 | 0.072 | 1E-06 | 0.029 | 1.37 | 1E-06 | 1.16 | -0.361 | 0.094 | 0.022 | 1E-06 | 1E-06 | -0.726 | 0.024 | 1.45 | -0.983 | 1.74 | -0.276 | 0.098 | -0.09 |
|  | HSA04910_INSULIN_SIGNALING_PATHWAY | 0.287 | 0.277 | 1E-06 | 0.084 | 0.802 | 1E-06 | 0.647 | -0.457 | 0.511 | 0.209 | 0.215 | 0.296 | 1E-06 | 0.119 | 0.531 | 1E-06 | 1.01 | -0.277 | 1.13 | -0.066 | 1E-06 | 0.398 | -0.538 | 0.112 | 0.482 | -0.394 | 1.15 | -0.848 | 1.16 | 0.304 |
|  | SIG_IL4RECEPTOR_IN_B_LYPHOCYTES | -0.05 | 0.146 | 0.139 | 0.026 | 0.875 | 0.193 | 0.781 | -0.041 | 0.751 | 0.02 | -0.316 | 0.123 | 0.003 | 0.573 | 0.364 | 0.02 | 0.536 | -0.381 | 1.36 | -0.417 | 1E-06 | 0.144 | -0.296 | 0.556 | 0.292 | -0.446 | 0.928 | -1.15 | 1.31 | 0.299 |
|  | ST_INTERLEUKIN_4_PATHWAY | -0.044 | 0.272 | 0.123 | 0.466 | 1.23 | 0.071 | 0.897 | 0.285 | 0.351 | 0.183 | 0.057 | 0.345 | 0.25 | 0.159 | 0.527 | 0.017 | 1.05 | -0.287 | 0.658 | -0.188 | 1E-06 | 0.349 | -0.509 | 0.138 | 0.353 | -0.403 | 1.18 | -1.04 | 0.728 | -0.135 |
|  | SIG_BCR_SIGNALING_PATHWAY | -0.101 | 0.144 | 0.044 | 0.659 | 1.16 | 0.019 | 0.929 | 0.806 | 0.44 | 0.045 | -0.087 | 0.174 | 0.001 | 0.145 | 0.618 | 1E-06 | 0.673 | -0.051 | 0.564 | -0.338 | 1E-06 | 0.285 | -0.438 | 0.137 | 0.303 | -0.456 | 0.698 | -0.979 | 0.531 | -0.088 |
|  | ST_B_CELL_ANTIGEN_RECEPTOR | -0.101 | 0.421 | 0.029 | 0.412 | 0.836 | 0.035 | 0.616 | 0.493 | 0.413 | 0.155 | -0.384 | 0.506 | 0.075 | 0.172 | 0.397 | 0.011 | 0.576 | -0.246 | 0.553 | 0.015 | -0.005 | 0.169 | -0.328 | 0.158 | 0.301 | -0.441 | 0.522 | -0.886 | 0.518 | -0.027 |
|  | ST_JNK_MAPK_PATHWAY | 0.259 | 0.141 | 1E-06 | 0.047 | 1.43 | -0.012 | 1.06 | 0.002 | 0.197 | 0.003 | 0.229 | 0.019 | 1E-06 | -0.016 | 0.618 | -0.043 | 1.26 | -0.087 | 0.133 | -0.509 | 1E-06 | 0.398 | 0.183 | -0.022 | 0.503 | 0.033 | 0.97 | -0.79 | 0.13 | -0.042 |
|  | CREBPATHWAY | 0.083 | 1E-06 | 0.023 | -0.047 | 1.21 | 0.05 | 0.773 | 0.189 | 0.09 | 0.241 | 0.06 | 1E-06 | 1E-06 | 0.283 | 0.593 | -0.002 | 0.907 | -0.038 | 0.127 | 1E-06 | 1E-06 | 0.104 | -0.341 | 0.263 | 0.462 | -0.148 | 1.12 | -0.04 | 0.13 | -0.005 |
|  | HSA00562_INOSITOL_PHOSPHATE_METABOLISM | -0.055 | 1E-06 | 0.002 | 0.69 | 0.98 | 0.003 | 0.918 | 0.464 | 0.415 | 0.07 | -0.407 | -0.02 | 1E-06 | -0.079 | 0.904 | 1E-06 | 0.617 | -0.038 | 0.094 | -0.107 | 1E-06 | 0.143 | -0.167 | -0.094 | 0.568 | -0.205 | 0.944 | -1.05 | 0.099 | -0.074 |
|  | HSA04662_B_CELL_RECEPTOR_SIGNALING_PATHWAY | -0.042 | 1E-06 | -0.051 | 0.815 | 0.954 | -0.037 | 0.705 | 0.506 | 0.145 | 0.07 | -0.382 | 1E-06 | 0.001 | 0.009 | 0.586 | 1E-06 | 0.327 | -0.086 | 0.095 | -0.12 | 0.002 | 1E-06 | -0.411 | 0.008 | 0.329 | -0.481 | 0.278 | -0.793 | 0.1 | -0.154 |
|  | PHOSPHATIDYLINOSITOL_SIGNALING_SYSTEM | -0.708 | 1E-06 | 0.166 | 0.301 | 0.992 | 0.198 | 0.785 | 0.569 | 0.34 | 0.397 | -0.333 | -0.028 | 0.039 | -0.084 | 0.594 | 1E-06 | 0.646 | -0.083 | 0.189 | -0.824 | 1E-06 | 1E-06 | -0.337 | -0.095 | 0.411 | -0.411 | 0.776 | -1.08 | 0.187 | -0.081 |
|  | HSA04070_PHOSPHATIDYLINOSITOL_SIGNALING_SYSTEM | -0.363 | 1E-06 | 1E-06 | 0.417 | 0.664 | 1E-06 | 0.616 | 0.109 | 0.124 | -0.025 | -0.441 | -0.034 | 1E-06 | -0.051 | 0.589 | -0.003 | 0.565 | -0.101 | 0.027 | -0.799 | 1E-06 | 0.011 | -0.38 | -0.049 | 0.33 | -0.398 | 0.573 | -1.23 | 0.027 | -0.263 |
| E | HSA04612_ANTIGEN_PROCESSING_AND_PRESENTATION | 0.13 | 1E-06 | 0.132 | 1.77 | -0.013 | 0.095 | -0.022 | 0.433 | 0.543 | -0.423 | 0.428 | -0.01 | -0.041 | 3.58 | -0.022 | 1E-06 | -0.05 | 0.37 | 1.24 | -1.29 | 1E-06 | 1E-06 | 0.057 | 4.03 | 0.052 | -0.435 | -0.038 | -0.036 | 1.25 | -0.272 |
|  | FASPATHWAY | 0.75 | 0.274 | 0.435 | 2.89 | 0.984 | 0.322 | 0.892 | 2.01 | 0.68 | 1.7 | 0.434 | 0.292 | 0.012 | 0.971 | 0.561 | -0.001 | 0.523 | 1.65 | 0.152 | 0.034 | 1E-06 | 0.164 | -0.26 | 0.968 | 0.503 | -0.019 | 0.639 | -0.162 | 0.157 | -0.235 |
|  | TNFR1PATHWAY | 0.79 | 0.178 | 0.418 | 2.66 | 0.79 | 0.261 | 0.763 | 1.07 | 0.414 | 0.83 | 0.355 | 0.201 | 1E-06 | 0.6 | 0.172 | 1E-06 | 0.201 | 0.11 | 0.068 | -0.018 | 1E-06 | 0.094 | -0.214 | 0.592 | 0.298 | -0.032 | 0.156 | -0.783 | 0.068 | -0.288 |
|  | CELLCYCLEPATHWAY | -0.243 | 0.486 | 0.135 | 2.42 | 0.187 | 0.204 | 0.147 | 2.98 | 0.913 | 1.22 | -0.061 | 0.406 | 1E-06 | 1.19 | 0.011 | -0.057 | -0.001 | 1.47 | 0.401 | 1E-06 | 1E-06 | 0.671 | -0.16 | 1.12 | 0.423 | -0.309 | 0.131 | 0.54 | 0.364 | 0.371 |
|  | G2PATHWAY | -0.019 | 0.391 | 0.172 | 1.31 | -0.006 | 0.38 | -0.024 | 2.03 | 0.564 | 3.08 | 0.108 | 0.446 | 1E-06 | 0.5 | 1E-06 | 1E-06 | -0.029 | 0.823 | 0.273 | 0.889 | 1E-06 | 0.23 | -0.48 | 0.484 | 0.212 | -0.346 | 0.071 | 1E-06 | 0.293 | 0.259 |
|  | HSA04115_P53_SIGNALING_PATHWAY | -0.02 | 0.273 | 0.413 | 2.19 | 1E-06 | 0.27 | -0.027 | 1.64 | 0.545 | 0.802 | -0.018 | 0.504 | 0.257 | 0.852 | -0.004 | 1E-06 | 1E-06 | 0.356 | 0.316 | 0.872 | 1E-06 | 0.106 | 0.005 | 0.882 | 0.089 | 0.008 | -0.043 | 0.054 | 0.307 | 0.413 |
|  | G1PATHWAY | -0.073 | 0.513 | 0.633 | 2.12 | 0.027 | 0.311 | -0.009 | 2.17 | 0.579 | 1.1 | -0.314 | 0.404 | 1E-06 | 0.16 | -0.001 | -0.042 | -0.048 | 0.801 | 0.05 | -0.154 | 1E-06 | 0.672 | -0.035 | 0.17 | -0.061 | 0.015 | -0.111 | 0.017 | 0.05 | -0.156 |
|  | DEATHPATHWAY | 0.062 | 0.418 | 0.136 | 2.73 | 0.341 | 0.27 | 0.534 | 0.003 | 0.978 | 1.16 | 0.452 | 0.439 | 0.105 | 0.571 | 0.176 | 0.416 | 0.252 | 0.012 | 0.603 | 1.78 | 1E-06 | 0.31 | -0.116 | 0.547 | 0.217 | -0.195 | 0.075 | -0.243 | 0.578 | 0.012 |
|  | ST_TUMOR_NECROSIS_FACTOR_PATHWAY | 1E-06 | 0.414 | 0.032 | 2.44 | 0.733 | 0.148 | 0.557 | 0.292 | 0.573 | 0.177 | 0.111 | 0.519 | 0.103 | 0.792 | 0.123 | 0.13 | 0.218 | 1E-06 | 0.877 | 0.186 | 1E-06 | 0.45 | 0.073 | 0.772 | 0.239 | 0.038 | 0.093 | -0.848 | 0.825 | -0.049 |
|  | APOPTOSIS_GENMAPP | 0.119 | 0.514 | 0.119 | 3.52 | 0.43 | 0.155 | 0.174 | 0.08 | 0.978 | 0.054 | 0.086 | 0.352 | 0.146 | 0.883 | 0.177 | 0.33 | 0.156 | 1E-06 | 0.508 | 0.21 | 1E-06 | 0.426 | 0.007 | 0.832 | 0.158 | -0.202 | 0.037 | -0.738 | 0.47 | -0.082 |
|  | APOPTOSIS | 0.068 | 0.294 | 0.133 | 3.56 | 0.54 | 0.051 | 0.373 | 0.741 | 0.544 | 0.106 | -0.024 | 0.336 | 0.011 | 0.681 | 0.155 | 0.012 | 0.057 | 1E-06 | 0.236 | -0.054 | 1E-06 | 0.235 | -0.175 | 0.673 | 0.158 | -0.419 | 0.027 | -0.783 | 0.226 | -0.301 |
|  | RASPATHWAY | NaN | 1.8 | 0.45 | 1.16 | 0.234 | 0.609 | 0.213 | 0.229 | 0.445 | 0.978 | NaN | 2.44 | 0.076 | 0.94 | 0.19 | 0.019 | 0.447 | -0.093 | 0.439 | 0.156 | NaN | 1.43 | -0.807 | 0.928 | -0.027 | -0.815 | 0.26 | -0.375 | 0.423 | -0.223 |
|  | GSK3PATHWAY | 0.762 | 0.433 | 0.447 | 0.394 | 0.224 | 0.149 | 0.253 | 0.066 | 0.484 | -0.231 | 0.111 | 0.636 | 0.906 | 0.982 | 0.396 | 0.542 | 0.689 | 0.234 | 0.652 | 0.415 | 1E-06 | 0.419 | 0.072 | 1.02 | 0.219 | 0.039 | 0.701 | 0.094 | 0.66 | 0.115 |
|  | RACCYCDPATHWAY | 0.05 | 0.417 | 0.075 | 0.761 | 0.452 | 0.159 | 0.273 | 0.668 | 0.938 | 1.13 | 0.024 | 0.75 | 1E-06 | 1.51 | 0.178 | 1E-06 | 0.208 | 1E-06 | 1.21 | 0.032 | 1E-06 | 0.41 | -0.203 | 1.44 | 0.156 | -0.417 | 0.101 | -0.09 | 1.16 | 0.346 |
|  | NKCELLSPATHWAY | NaN | 0.136 | 0.131 | 0.446 | 0.324 | 0.426 | 0.221 | 0.681 | 0.281 | 0.003 | NaN | 0.03 | 0.105 | 1.69 | 0.205 | 0.191 | 0.158 | -0.089 | 0.157 | -0.032 | NaN | 0.001 | -0.173 | 1.77 | 0.196 | -0.358 | 0.114 | -0.78 | 0.17 | -0.293 |
|  | HSA00030_PENTOSE_PHOSPHATE_PATHWAY | 0.13 | 0.508 | 0.021 | 2.82 | 0.149 | 0.151 | 0.165 | 0.739 | 0.252 | 1.72 | 0.456 | 1.07 | 1E-06 | 0.406 | 0.194 | 1E-06 | 0.386 | 2.03 | 0.582 | 2.33 | -0.001 | 0.84 | -0.068 | 0.382 | 0.001 | 0.011 | 0.102 | 0.238 | 0.549 | 0.623 |
|  | PENTOSE_PHOSPHATE_PATHWAY | NaN | 0.466 | 0.023 | 2.12 | 0.024 | 0.162 | 0.063 | 0.527 | 0.447 | 0.944 | NaN | 1.12 | 0.043 | 0.369 | 0.113 | 1E-06 | 0.431 | 2.04 | 1.02 | 1.7 | NaN | 1.09 | -0.035 | 0.374 | 0.002 | -0.024 | 0.032 | 0.346 | 0.966 | 0.49 |
|  | HSA00530_AMINOSUGARS_METABOLISM | 0.521 | 0.26 | 0.024 | 3.09 | 0.1 | 1E-06 | 0.06 | 0.745 | 0.069 | 0.886 | 0.595 | 0.302 | 0.073 | 0.03 | 0.04 | 1E-06 | 0.248 | 2.15 | 0.08 | 2.78 | 1E-06 | 0.333 | 0.045 | 0.026 | -0.04 | 0.146 | 0.058 | -0.026 | 0.087 | 0.001 |
|  | HSA01030_GLYCAN_STRUCTURES_BIOSYNTHESIS_1 | 0.18 | 0.159 | 0.169 | 0.523 | 1E-06 | 0.107 | -0.032 | 0.822 | 0.447 | 0.073 | 0.029 | 0.152 | 0.371 | -0.441 | 0.091 | 0.409 | -0.015 | 1.19 | 0.841 | 1.06 | 1E-06 | 1E-06 | 0.063 | -0.46 | 0.151 | 0.22 | 0.074 | -0.06 | 0.811 | 0.265 |
|  | HSA00600_SPHINGOLIPID_METABOLISM | 0.554 | 0.005 | 0.125 | 1.91 | 0.777 | 0.064 | 0.547 | 0.282 | 0.335 | -0.106 | 0.437 | -0.003 | 0.093 | 0.1 | 0.728 | 1E-06 | 0.578 | 0.967 | 0.453 | 1.3 | 1E-06 | 1E-06 | 0.071 | 0.127 | 0.33 | 0.214 | 0.368 | -0.036 | 0.502 | -0.027 |
|  | HSA00052_GALACTOSE_METABOLISM | 0.815 | 0.151 | 1E-06 | 1.52 | 1E-06 | 1E-06 | -0.026 | 0.147 | 0.005 | 0.207 | 0.726 | 0.212 | 0.195 | 0.032 | -0.011 | 1E-06 | -0.019 | 0.943 | 0.318 | 2.19 | 1E-06 | 0.167 | 0.19 | 0.027 | -0.03 | 0.164 | -0.013 | 0.016 | 0.361 | 0.132 |
|  | HSA00051_FRUCTOSE_AND_MANNOSE_METABOLISM | 0.125 | 0.067 | -0.037 | 1.31 | 1E-06 | -0.347 | 1E-06 | 0.374 | -0.041 | 0.107 | 0.211 | 0.243 | 0.004 | 0.319 | 0.024 | -0.103 | 0.054 | 0.82 | 0.091 | 1.64 | 1E-06 | 0.141 | 0.123 | 0.305 | -0.024 | 0.223 | 1E-06 | -0.194 | 0.097 | 0.023 |
|  | HSA04920_ADIPOCYTOKINE_SIGNALING_PATHWAY | 0.012 | 0.255 | -0.29 | -0.121 | 0.34 | -0.62 | 0.38 | -1.47 | 0.372 | -0.848 | 1E-06 | 0.299 | 0.106 | 0.646 | 0.29 | 1E-06 | 0.532 | -0.092 | 1.43 | 0.208 | 1E-06 | 1E-06 | 0.472 | 0.689 | 0.238 | 0.219 | 0.323 | -0.352 | 1.37 | 0.866 |
|  | HSA00561_GLYCEROLIPID_METABOLISM | 0.066 | 0.003 | 1E-06 | -0.197 | 0.086 | -0.053 | 0.248 | -0.253 | 0.008 | -1.21 | 0.13 | 1E-06 | 1E-06 | 0.611 | 0.618 | -0.003 | 0.555 | 0.037 | 0.099 | 0.908 | 1E-06 | 1E-06 | -0.18 | 0.612 | 0.452 | -0.035 | 0.386 | 0.063 | 0.111 | 0.365 |
|  | NUCLEAR_RECEPTORS | 0.047 | 0.11 | -0.081 | -1.31 | 0.139 | -0.271 | 0.123 | -1.28 | -0.083 | -1.51 | 0.733 | 0.173 | 0.197 | 0.124 | 0.364 | 0.021 | 0.229 | -0.054 | 0.377 | -0.305 | 1E-06 | 1E-06 | 0.554 | 0.126 | 0.305 | 0.247 | 0.387 | -0.022 | 0.386 | 0.678 |
|  | HSA00590_ARACHIDONIC_ACID_METABOLISM | -1.24 | -0.067 | -0.53 | -0.137 | -0.011 | -0.595 | -0.022 | -0.812 | 0.061 | -1.5 | 0.038 | -0.013 | 1E-06 | 0.407 | 1E-06 | 1E-06 | -0.015 | 0.253 | 0.336 | 0.53 | 1E-06 | -0.567 | 0.163 | 0.393 | -0.057 | 0.03 | -0.035 | 0.806 | 0.323 | 0.698 |
|  | HSA04510_FOCAL_ADHESION | 0.01 | 0.073 | 0.053 | 0.339 | -0.001 | 0.082 | -0.024 | 0.31 | 0.332 | 0.028 | 0.03 | 0.2 | -0.144 | -0.343 | -0.021 | 1E-06 | -0.02 | -0.232 | 0.45 | -0.236 | 1E-06 | 0.2 | -0.164 | -0.342 | -0.14 | -0.432 | 0.145 | -2.33 | 0.449 | -1.6 |
|  | INTEGRIN_MEDIATED_CELL_ADHESION_KEGG | -0.043 | 0.124 | 1E-06 | 0.441 | -0.003 | 0.05 | -0.038 | 0.252 | 0.45 | 0.177 | -0.082 | 0.181 | -0.154 | -0.633 | -0.003 | -0.028 | -0.034 | -0.232 | 0.239 | -0.069 | 1E-06 | 0.107 | -0.292 | -0.74 | -0.048 | -0.336 | -0.03 | -2.67 | 0.248 | -1.19 |
|  | HSA04512_ECM_RECEPTOR_INTERACTION | -0.157 | -0.008 | 1E-06 | -0.071 | -0.179 | -0.039 | -0.532 | 0.134 | 0.036 | -0.788 | 0.132 | -0.024 | -0.176 | -1.39 | -0.46 | -0.027 | -0.399 | -0.096 | 0.23 | -0.197 | 1E-06 | 1E-06 | 0.745 | -1.46 | -1.16 | 0.288 | -0.548 | -1.16 | 0.222 | -1.74 |
|  | HSA04350_TGF_BETA_SIGNALING_PATHWAY | 0.29 | 0.4 | -0.066 | 0.951 | 0.288 | 0.033 | -0.053 | 1E-06 | 0.373 | 0.202 | 0.355 | 0.634 | -0.176 | 0.041 | 0.181 | -0.034 | -0.016 | -0.216 | 0.342 | 0.053 | 1E-06 | 1E-06 | -0.165 | 0.047 | 0.425 | 0.029 | 0.121 | -0.324 | 0.334 | -0.152 |
|  | HSA04530_TIGHT_JUNCTION | 0.061 | 0.516 | 0.372 | 0.095 | 1E-06 | 0.244 | -0.031 | 0.293 | 0.1 | -0.085 | 0.446 | 0.583 | 0.1 | 1E-06 | -0.006 | 0.118 | -0.004 | 1E-06 | 0.241 | -0.129 | 1E-06 | 0.411 | 0.065 | 1E-06 | 0.087 | 0.055 | 0.261 | -0.426 | 0.247 | -0.234 |
|  | ST_GAQ_PATHWAY | 0.006 | 0.429 | 1E-06 | 0.069 | -0.009 | 1E-06 | -0.026 | 0.035 | 0.539 | -0.031 | 0.133 | 0.61 | 1E-06 | 0.117 | 0.235 | -0.011 | 0.055 | -0.088 | 0.547 | 0.002 | 1E-06 | 0.298 | 0.054 | 0.123 | -0.035 | -0.198 | -0.001 | -1.03 | 0.571 | -0.086 |
|  | ALKPATHWAY | -0.088 | 0.036 | 0.424 | 0.308 | -0.021 | 0.208 | -0.116 | 1E-06 | 0.338 | -0.122 | -0.319 | 0.098 | 0.085 | 0.149 | -0.014 | 0.122 | -0.031 | -0.08 | 0.083 | -0.007 | -0.001 | 1E-06 | 1E-06 | 0.138 | 0.005 | -0.225 | -0.031 | 0.002 | 0.089 | -0.075 |
|  | HSA01031_GLYCAN_STRUCTURES_BIOSYNTHESIS_2 | -0.267 | 1E-06 | 1E-06 | 0.417 | 1E-06 | 1E-06 | -0.035 | 0.677 | 0.225 | -0.292 | -0.592 | -0.042 | 0.079 | 0.041 | 0.065 | 1E-06 | -0.02 | 0.338 | 0.114 | -0.18 | -0.082 | -0.355 | -0.034 | 0.036 | 0.216 | -0.272 | 0.119 | 0.333 | 0.115 | -0.115 |
|  | PROSTAGLANDIN_AND_LEUKOTRIENE_METABOLISM | -0.703 | 1E-06 | -0.063 | 0.087 | -0.01 | 1E-06 | -0.049 | 0.02 | 0.359 | -1.82 | 0.06 | -0.032 | 0.001 | 0.037 | -0.012 | 0.001 | -0.017 | 0.237 | 0.185 | -0.336 | 1E-06 | 1E-06 | 0.006 | 0.036 | -0.042 | -0.349 | 1E-06 | 0.345 | 0.188 | 0.261 |
|  | ST_WNT_BETA_CATENIN_PATHWAY | 0.036 | -0.124 | 0.627 | 0.025 | 0.01 | 0.785 | 1E-06 | 0.006 | 0.005 | -0.118 | -0.057 | -0.025 | 0.493 | 0.028 | 0.041 | 0.541 | 0.339 | -0.038 | 1E-06 | -0.151 | 1E-06 | -0.207 | -0.165 | 0.028 | -0.296 | 0.228 | 1E-06 | -0.285 | 1E-06 | -0.14 |
|  | GLYCEROPHOSPHOLIPID_METABOLISM | -0.069 | 1E-06 | 0.193 | 0.027 | 0.168 | 0.028 | 0.034 | -0.009 | 0.021 | -0.604 | 0.209 | -0.032 | 0.386 | -0.446 | 0.516 | 0.021 | 0.326 | -0.09 | -0.017 | -0.111 | 1E-06 | 1E-06 | 0.074 | -0.47 | 0.456 | 1E-06 | 0.503 | -0.098 | 1E-06 | -0.043 |
|  | NFATPATHWAY | 0.05 | 0.123 | 1E-06 | 0.02 | -0.011 | 1E-06 | 0.098 | -0.043 | 0.142 | -0.105 | 0.022 | 1E-06 | 1E-06 | 0.03 | 0.44 | -0.023 | 0.228 | -0.041 | 0.162 | -0.451 | 1E-06 | 0.139 | -0.73 | 0.024 | 0.155 | -0.485 | 0.297 | -0.126 | 0.167 | -0.043 |
|  | HSA04310_WNT_SIGNALING_PATHWAY | -0.19 | 1E-06 | 0.03 | 0.439 | 1E-06 | 0.014 | -0.044 | 0.28 | 0.134 | 0.161 | -0.232 | -0.016 | 1E-06 | -0.239 | -0.015 | -0.007 | -0.036 | -0.083 | 0.154 | -0.325 | 1E-06 | 1E-06 | -0.5 | -0.208 | 0.237 | 0.045 | 0.108 | -0.092 | 0.144 | 0.019 |
|  | HSA04720_LONG_TERM_POTENTIATION | -0.196 | 0.149 | 0.029 | 0.122 | -0.003 | 0.062 | -0.001 | 0.174 | 0.13 | -0.177 | -0.159 | -0.01 | 1E-06 | -0.157 | 0.311 | -0.009 | -0.011 | -0.221 | 0.151 | -0.446 | 1E-06 | 1E-06 | -0.489 | -0.145 | 0.331 | -0.223 | 0.321 | -0.017 | 0.148 | -0.077 |
|  | SIG_CHEMOTAXIS | 0.047 | 0.095 | 0.056 | 0.402 | 0.101 | 0.252 | -0.008 | 0.337 | 0.19 | 0.543 | -0.085 | 0.187 | 1E-06 | 0.069 | -0.015 | 1E-06 | -0.035 | -0.169 | 0.139 | -0.886 | 1E-06 | 1E-06 | -0.095 | 0.061 | 0.136 | 0.035 | 0.063 | -0.314 | 0.132 | -0.429 |
|  | KERATINOCYTEPATHWAY | 0.112 | 0.092 | 1E-06 | 0.417 | 0.382 | 1E-06 | 0.191 | 0.279 | 0.089 | 0.078 | 0.123 | -0.003 | 1E-06 | 0.035 | 0.167 | 1E-06 | 0.325 | -0.093 | 0.134 | -0.115 | 1E-06 | -0.025 | 0.171 | 0.026 | 0.198 | -0.228 | 0.172 | -0.368 | 0.13 | -0.287 |
|  | ST_GA12_PATHWAY | 0.106 | 1E-06 | 0.066 | 0.009 | 0.128 | -0.238 | 0.004 | 0.46 | 0.009 | 0.184 | 0.336 | -0.047 | 0.018 | -0.164 | -0.015 | -0.002 | -0.018 | -0.187 | 1E-06 | -0.005 | 1E-06 | -0.186 | 0.189 | -0.166 | 0.156 | 0.212 | 0.171 | -0.575 | 1E-06 | -0.238 |
|  | G_PROTEIN_SIGNALING | -0.087 | 0.197 | 0.131 | 0.228 | 0.513 | 0.11 | 0.217 | 0.311 | 0.148 | -0.323 | 0.036 | 0.129 | 1E-06 | -0.21 | 0.292 | -0.015 | 0.312 | -0.092 | 0.128 | -1.17 | 1E-06 | 0.353 | 0.036 | -0.214 | 0.058 | 0.008 | 0.118 | -0.45 | 0.13 | -0.264 |
|  | HSA04912_GNRH_SIGNALING_PATHWAY | -0.062 | -0.058 | 1E-06 | 0.082 | 0.256 | -0.032 | 0.261 | 0.29 | 0.009 | -0.312 | 0.045 | -0.035 | 1E-06 | -0.432 | 0.177 | -0.005 | 0.259 | -0.342 | -0.002 | -1.36 | 1E-06 | 1E-06 | -0.102 | -0.481 | 0.192 | 0.05 | 0.377 | -0.455 | -0.007 | -0.545 |
|  | PROSTAGLANDIN_SYNTHESIS_REGULATION | NaN | 0.247 | -0.614 | 0.948 | 1E-06 | -0.839 | -0.015 | 0.482 | 0.538 | -0.182 | NaN | 0.016 | -0.13 | -0.18 | -0.171 | -0.398 | -0.015 | 1E-06 | 0.966 | -0.426 | NaN | 0.336 | -0.146 | -0.176 | -0.921 | -0.47 | -0.618 | -0.577 | 0.968 | -0.116 |
|  | WNT_SIGNALING | -0.983 | -0.319 | 0.132 | 0.404 | -0.022 | 0.047 | -0.022 | 0.606 | 0.128 | -0.032 | -0.323 | -0.327 | 0.097 | -0.074 | -0.483 | 0.019 | -0.014 | -0.085 | 0.081 | -0.991 | 1E-06 | -0.203 | 0.017 | -0.086 | -0.611 | 0.145 | -0.331 | -0.205 | 0.081 | -0.177 |
|  | HSA04010_MAPK_SIGNALING_PATHWAY | -0.064 | 0.199 | 0.022 | 0.132 | -0.026 | 0.045 | -0.093 | 0.228 | 0.003 | -0.314 | -0.328 | 1E-06 | 1E-06 | -0.383 | -0.175 | 1E-06 | -0.006 | -0.188 | -0.231 | -0.807 | 1E-06 | 1E-06 | 0.053 | -0.409 | -0.133 | -0.277 | -0.057 | -0.662 | -0.265 | -0.38 |
|  | HSA04360_AXON_GUIDANCE | -0.248 | -0.067 | -0.068 | 0.161 | -0.047 | -0.033 | -0.148 | 0.263 | 0.006 | 0.082 | -0.44 | -0.03 | 1E-06 | -0.541 | -0.278 | -0.06 | -0.363 | -0.169 | -0.047 | -0.5 | 1E-06 | -0.169 | 0.04 | -0.541 | -0.16 | 0.045 | -0.157 | -0.77 | -0.048 | -0.558 |
|  | HSA04340_HEDGEHOG_SIGNALING_PATHWAY | -0.336 | 1E-06 | 1E-06 | -0.434 | -0.244 | 1E-06 | -0.17 | -0.076 | -0.327 | -1.87 | -0.144 | -0.019 | -0.142 | -0.231 | -0.485 | -0.042 | -0.194 | -0.204 | -0.321 | -0.83 | 1E-06 | 1E-06 | -0.478 | -0.296 | -0.339 | -0.318 | -0.575 | -0.161 | -0.327 | -0.277 |
|  | TCRPATHWAY | -0.037 | 0.179 | -0.089 | 0.776 | 0.277 | -0.034 | 0.266 | 0.038 | 0.188 | 0.026 | -0.533 | 0.128 | -0.171 | 0.399 | 0.059 | -0.174 | -0.022 | -0.321 | 0.17 | -2 | 1E-06 | 0.272 | -0.654 | 0.395 | 0.154 | -0.785 | 0.075 | -0.854 | 0.188 | -0.049 |
|  | CALCINEURIN_NF_AT_SIGNALING | -0.662 | 1E-06 | 0.011 | 0.575 | 0.284 | -0.005 | 0.088 | 0.685 | 0.009 | -0.123 | -0.411 | -0.024 | -0.157 | 1E-06 | 0.16 | -0.066 | -0.002 | -0.233 | -0.071 | -2.51 | 1E-06 | -0.164 | -0.485 | 1E-06 | 0.448 | -0.431 | 0.467 | -0.285 | -0.114 | -0.283 |
|  | HSA04660_T_CELL_RECEPTOR_SIGNALING_PATHWAY | -0.201 | -0.407 | 0.029 | 1.1 | 0.753 | 0.032 | 0.636 | 0.839 | 0.048 | 0.287 | -0.866 | -0.627 | 1E-06 | 0.009 | 0.115 | -0.007 | 1E-06 | -0.171 | -0.011 | -1.98 | 1E-06 | -0.587 | -0.491 | 0.008 | 0.316 | -1.01 | 0.228 | -1.16 | -0.02 | -0.287 |
|  | HSA04630_JAK_STAT_SIGNALING_PATHWAY | -0.023 | -0.123 | -0.666 | 0.111 | -0.002 | -0.947 | -0.03 | 0.066 | 0.004 | -1.88 | -0.132 | -0.322 | -0.173 | -0.065 | -0.018 | -0.114 | -0.004 | -0.173 | -0.04 | -1.29 | 1E-06 | -0.209 | -0.323 | -0.072 | -0.057 | -0.462 | -0.041 | -1.27 | -0.027 | -0.629 |
|  | DCPATHWAY | NaN | -0.364 | -0.652 | 0.026 | 0.023 | -0.62 | 1E-06 | 0.231 | -0.742 | -1.65 | NaN | -0.511 | -0.327 | -0.006 | -0.006 | -0.39 | -0.041 | -0.055 | -0.513 | -2.54 | NaN | -0.642 | -0.511 | -0.005 | -0.003 | -0.775 | 1E-06 | -0.192 | -0.491 | -0.376 |
| F | HSA04640_HEMATOPOIETIC_CELL_LINEAGE | -0.328 | -0.303 | -0.799 | 0.468 | 1E-06 | -0.648 | -0.092 | 0.661 | -0.714 | -3.18 | -0.521 | -0.532 | -0.9 | -0.46 | -0.638 | -0.609 | -0.417 | 1E-06 | -0.78 | -1.62 | 1E-06 | -0.189 | -0.167 | -0.489 | -1.08 | -0.924 | -0.59 | -0.214 | -0.699 | -0.558 |
|  | HSA04060_CYTOKINE_CYTOKINE_RECEPTOR_INTERACTION | -0.724 | -0.342 | -0.219 | 0.329 | -0.092 | -0.253 | -0.266 | 0.312 | -1.56 | -3.82 | -0.889 | -0.752 | -0.154 | -0.527 | -0.456 | -0.039 | -0.262 | -0.055 | -1.13 | -2.05 | 1E-06 | -0.693 | -0.159 | -0.52 | -0.377 | -0.742 | -0.467 | -0.356 | -1.17 | -1.89 |
|  | HSA04020_CALCIUM_SIGNALING_PATHWAY | -0.656 | -0.424 | -1.51 | -0.719 | -0.684 | -1.71 | -0.703 | -0.251 | -0.506 | -5 | -0.451 | -1.26 | -0.34 | -0.959 | -0.759 | -0.592 | -0.526 | -0.175 | -0.455 | -2.89 | 1E-06 | -0.815 | -0.162 | -0.857 | -0.963 | -0.329 | -0.565 | 0.057 | -0.507 | -0.37 |
|  | GPCRDB_OTHER | -0.19 | -0.775 | -1.08 | -0.304 | -0.194 | -0.65 | -0.164 | -0.156 | -1.68 | -3.13 | -0.532 | -0.783 | -0.338 | -0.644 | -0.695 | -0.07 | -1.22 | -0.224 | -1.64 | -2.96 | 1E-06 | -0.65 | -0.012 | -0.73 | -0.932 | -0.398 | -0.654 | -0.278 | -1.59 | -0.288 |
|  | MONOAMINE_GPCRS | NaN | -0.335 | -1.43 | 2 | -0.439 | -0.928 | -0.724 | -0.764 | -1.83 | -5 | NaN | -0.526 | -0.298 | -0.866 | -0.466 | -0.572 | -0.399 | -0.093 | -1.67 | -2.77 | NaN | -0.58 | -0.249 | -0.876 | -0.903 | -0.245 | -0.622 | 0.003 | -1.93 | -0.235 |
|  | HSA01430_CELL_COMMUNICATION | 0.075 | -0.397 | -0.296 | -0.587 | -3.16 | -0.032 | -5 | -0.075 | -0.124 | -2.1 | 0.063 | -0.535 | -0.947 | -1.68 | -2.92 | -0.399 | -1.75 | -0.085 | -0.175 | -1.57 | 1E-06 | -0.457 | 0.087 | -1.6 | -2.55 | 0.029 | -2.67 | -0.285 | -0.152 | -2.17 |
|  | PEPTIDE_GPCRS | -1.12 | -3.16 | -3.09 | -1.04 | -0.51 | 2 | -0.577 | -0.599 | -1.71 | -5 | -0.87 | -3.04 | -3.01 | -1.55 | -0.735 | -1.59 | -0.567 | -0.39 | -1.71 | -5 | 1E-06 | -2.86 | 0.037 | -1.63 | -1.25 | 0.034 | -1.13 | -0.001 | -1.76 | -0.74 |
|  | GPCRDB_CLASS_A_RHODOPSIN_LIKE | -1.08 | -1.16 | -3.17 | -1.68 | -3.11 | -3.61 | -3.14 | -0.668 | -2.35 | -5 | -1.12 | -1.9 | -3.16 | -1.24 | -3.04 | -2.45 | -3.58 | -0.277 | -2.5 | -5 | 1E-06 | -1.58 | -0.177 | -1.22 | -3.05 | -0.34 | -3.17 | 1E-06 | -2.64 | -1.16 |
|  | HSA04080_NEUROACTIVE_LIGAND_RECEPTOR_INTERACTION | -3.18 | -2.27 | -5 | -3.36 | -5 | 2 | -5 | -1.62 | -5 | -5 | -1.85 | -5 | -2.98 | -1.79 | -5 | -3.03 | -5 | -0.362 | -5 | -5 | 1E-06 | -3.27 | -0.5 | -1.55 | -5 | -0.426 | -5 | -0.038 | -5 | -1.82 |
